# Dimethyl fumarate modulates the dystrophic disease program following short-term treatment

**DOI:** 10.1172/jci.insight.165974

**Published:** 2023-11-08

**Authors:** Cara A. Timpani, Stephanie Kourakis, Danielle A. Debruin, Dean G. Campelj, Nancy Pompeani, Narges Dargahi, Angelo P. Bautista, Ryan M. Bagaric, Elya J. Ritenis, Lauren Sahakian, Didier Debrincat, Nicole Stupka, Patricia Hafner, Peter G. Arthur, Jessica R. Terrill, Vasso Apostolopoulos, Judy B. de Haan, Nuri Guven, Dirk Fischer, Emma Rybalka

**Affiliations:** 1 Institute for Health and Sport (IHeS), Victoria University, Melbourne, Victoria, Australia.; 2 Australian Institute for Musculoskeletal Science (AIMSS), St Albans, Victoria, Australia.; 3 Department of Medicine – Western Health, Melbourne Medical School, The University of Melbourne, St Albans, Victoria, Australia.; 4 Florey Institute of Neuroscience and Mental Health, Heidelberg, Victoria, Australia.; 5 School of Molecular Sciences, The University of Western Australia, Perth, Western Australia, Australia.; 6 College of Health and Biomedicine, Victoria University, Melbourne, Victoria, Australia.; 7 Division of Neuropaediatrics and Developmental Medicine, University Children’s Hospital of Basel (UKBB), Basel, Switzerland.; 8 Basic Science Domain, Oxidative Stress Laboratory, Baker Heart and Diabetes Institute, Melbourne, Victoria, Australia.; 9 Department of Immunology and Pathology, Central Clinical School, Monash University, Melbourne, Victoria, Australia.; 10 Department of Physiology, Anatomy and Microbiology, La Trobe University, Melbourne, Victoria, Australia.; 11 Faculty of Science, Engineering and Technology, Swinburne University, Melbourne, Victoria, Australia.; 12 Baker Department of Cardiometabolic Health, University of Melbourne, Parkville, Victoria, Australia.; 13 School of Pharmacy and Pharmacology, University of Tasmania, Hobart, Tasmania, Australia.

**Keywords:** Muscle Biology, Therapeutics, Drug therapy, Neuromuscular disease, Skeletal muscle

## Abstract

New medicines are urgently required to treat the fatal neuromuscular disease Duchenne muscular dystrophy (DMD). Dimethyl fumarate (DMF) is a potent immunomodulatory small molecule nuclear erythroid 2-related factor 2 activator with current clinical utility in the treatment of multiple sclerosis and psoriasis that could be effective for DMD and rapidly translatable. Here, we tested 2 weeks of daily 100 mg/kg DMF versus 5 mg/kg standard-care prednisone (PRED) treatment in juvenile *mdx* mice with early symptomatic DMD. Both drugs modulated seed genes driving the DMD disease program and improved force production in fast-twitch muscle. However, only DMF showed pro-mitochondrial effects, protected contracting muscles from fatigue, improved histopathology, and augmented clinically compatible muscle function tests. DMF may be a more selective modulator of the DMD disease program than PRED, warranting follow-up longitudinal studies to evaluate disease-modifying impact.

## Graphical abstract

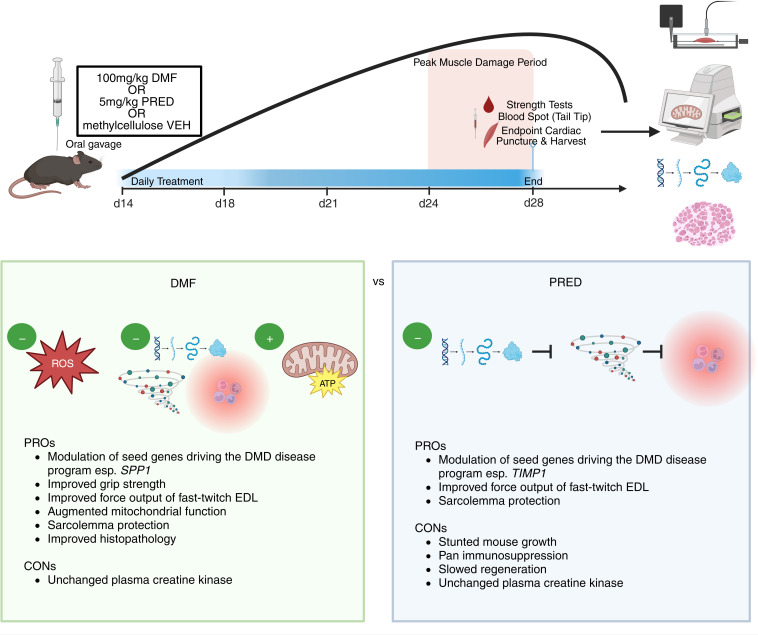

## Introduction

Drug repurposing is an efficient strategy to deliver medicines to market in a time- and cost-effective manner. Rare diseases could benefit most from this strategy because they are often fatal, are rapidly progressive, and have high unmet clinical need (1). Duchenne muscular dystrophy (DMD) is a devastating myogenic disease that matches these criteria, making it a good candidate for drug repurposing. In DMD, muscles lack functional dystrophin protein from the cytoskeleton due to mutation of the longest human gene, *DMD*. This deficiency results in muscle fragility, dysregulated ion channels, and a complex pathophysiology leading to the chronic degeneration of skeletal muscles (reviewed in ref. 2). Cardiac and smooth muscle are also affected, as well as other tissue types expressing dystrophin isoforms (e.g., vascular endothelium, brain) but to a lesser extent. Patients with DMD rely on wheelchairs by approximately 12 years (3, 4) and ultimately die from cardiorespiratory failure in early adulthood (~26 years) (4). Corticosteroids (i.e., prednisone/prednisolone [PRED], deflazacort) have prevailed as standard-care pharmacotherapy for more than 20 years, delaying loss of ambulation by 2–3 years and considerably reducing the requirement for spinal corrective surgery and mechanical ventilation and the risk of cardiomyopathy (5). Their chronic use, however, is associated with extensive side effects, including metabolic dysregulation leading to excessive weight gain/obesity, stunted growth, and osteoporosis, making them unsuitable for some patients (6). Emerging therapeutics targeted at the genetic mechanism offer new hope particularly for younger patients who are still ambulatory (7). However, maximum efficacy may depend on multimodal treatments that also target the underlying pathobiology. There remain few treatment options for patients with advanced DMD.

**Figure 1:**
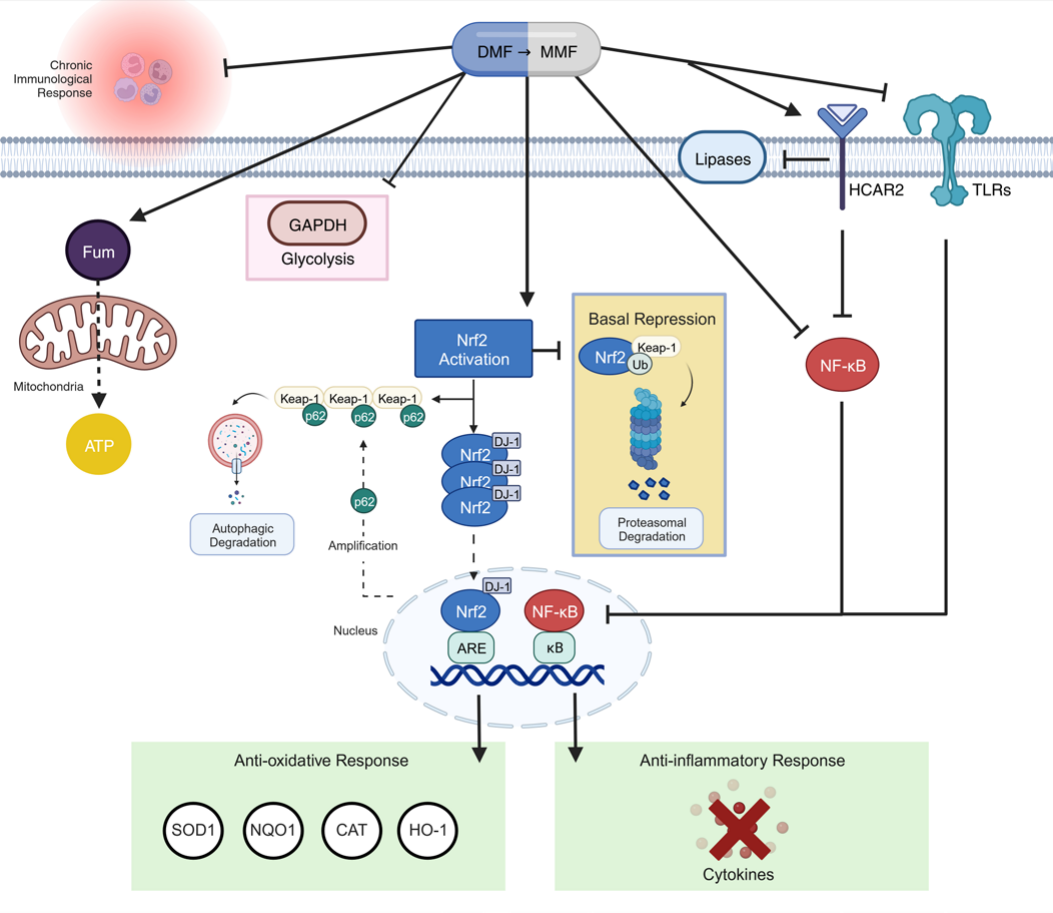
Mechanisms of action of DMF. DMF is rapidly converted to bioactive monomethyl fumarate (MMF) in the gut and circulated to tissues. Inside cells, MMF is converted to fumarate, which binds kelch-like ECH-associated protein 1 (Keap1), resulting in dissociation of the Keap1-Nrf2 complex. Keap1 represses Nrf2 activity by targeting the complex for degradation by the ubiquitin proteosome. Once dissociated from Keap1, DJ-1 chaperones Nrf2 into the nucleus, where Nrf2 binds the antioxidant response element (ARE), initiating transcription of antioxidant genes superoxide dismutase 1 (SOD1), NAD(P)H dehydrogenase:quinone oxidoreductase (NQO1), catalase (CAT), and hemoxygenase-1 (HO-1). Meanwhile, Keap1 is sequestered by p62, which initiates autophagy and amplifies Nrf2-mediated ARE transcription. Fumarate also inhibits master inflammation regulator, nuclear factor κB (NF-κB), which suppresses nuclear binding of κB and transcription of pro-inflammatory cytokines. MMF also inhibits NF-κB via agonism of the hydroxycarboxylic acid receptor 2 (HCAR2) and antagonism of Toll-like receptors (TLRs) on the membrane. Fumarate causes metabolic shifts by inhibiting glyceraldehyde-3-phosphate dehydrogenase (GAPDH) activity, and therefore, glycolysis. Fumarate enters mitochondria via the malate-aspartate shuttle, where it is ultimately sequestered into the matrix Krebs cycle and is completely metabolized to yield ATP and CO_2_.

Dimethyl fumarate (DMF) is a small molecule immunomodulator clinically used to treat relapsing remitting multiple sclerosis (RRMS; Tecfidera) and psoriasis (Fumaderm). Both diseases are driven by autoimmunity and DMF effectively treats this etiology. Alternative therapeutic applications are currently under investigation for chronic diseases that share similar etiology in both clinical and preclinical studies (reviewed in ref. 8). DMF’s established mechanism of action (MOA) is through activation of the transcription factor, nuclear erythroid factor-2 related factor 2 (Nrf2), which incites the cytoprotective program against toxic stress (summarized in Figure 1). This program results in antioxidation, antiinflammation, and detoxification and is especially effective in the immune system to control overactivation. Additional complementary mechanisms (shown in Figure 1) include: (i) agonism of HCAR2, to inhibit membrane breakdown and resolve inflammation (9); (ii) inhibition of the glycolytic enzyme GAPDH, which rewires metabolism through the mitochondria (10); and (iii) blocking Toll-like receptor 4 induction of pro-inflammatory cytokines (11). Following oral consumption, DMF is rapidly converted in the gut to its bioactive form, MMF, which is taken up by cells and converted to fumarate (for multispecies pharmacokinetic and pharmacodynamic profiles, see ref. 12). Fumarate causes succinylation of key cytosolic proteins, including negative repressor of Nrf2, Keap1, resulting in Nrf2 activation (13) and consequently upregulation of NQO1, a robust pharmacodynamic biomarker of DMF’s MOA (12). It also stimulates mitochondrial oxidative phosphorylation through integration into the Krebs cycle, where it is ultimately completely metabolized (14).

Investigational therapeutics for DMD have historically fallen short in clinical trials (15), highlighting both the complexity of the pathophysiological milieu that drives degeneration of dystrophin-deficient muscles and disparity between the human condition and animal models. Preclinical drug investigations primarily use the *mdx* mouse, which recapitulates a milder DMD phenotype than human patients (16), resulting in poor translation of experimental treatments into the clinic. Despite inducing robust muscle preservation in preclinical *mdx* trials using sexually mature, disease-stable mice, promising drugs have so far been unsuccessful in attenuating disease progression in clinical trials (reviewed in ref. 17, using myostatin inhibitors as an example). Severe damage bouts during the juvenile period and established pathology in the senile period in *mdx* mice could, and should, be leveraged to assess more human-comparable disease.

We recently developed a theoretical context for Nrf2’s suitability as a candidate drug target to treat DMD (18). Mitochondrial function, autophagy, satellite cell cycling, calcium homeostasis, and inflammation are all chronically dysregulated in DMD, and once activated, Nrf2 can positively modulate these processes to promote cell survival. Indeed, knocking Nrf2 out of *mdx* mice escalates DMD pathology when disease is aggravated by running (19), and applying Nrf2 activators sulforaphane (20) and curcumin (21) to the *mdx* mouse lessens myopathy. However, no study has investigated an Nrf2 activator drug with clinical indication that could be rapidly translated for DMD or contrasted drug efficacy against standard-care glucocorticoids. In this proof-of-concept study, we aimed to evaluate the efficacy of DMF against standard-care PRED using juvenile *mdx* mice with severe spontaneous-onset MD. Our data demonstrate DMF as a translational candidate for more comprehensive preclinical evaluation.

## Results

### DMF is well tolerated in mice and improves muscle function test performance but not blood biomarkers of DMD.

**Figure 2:**
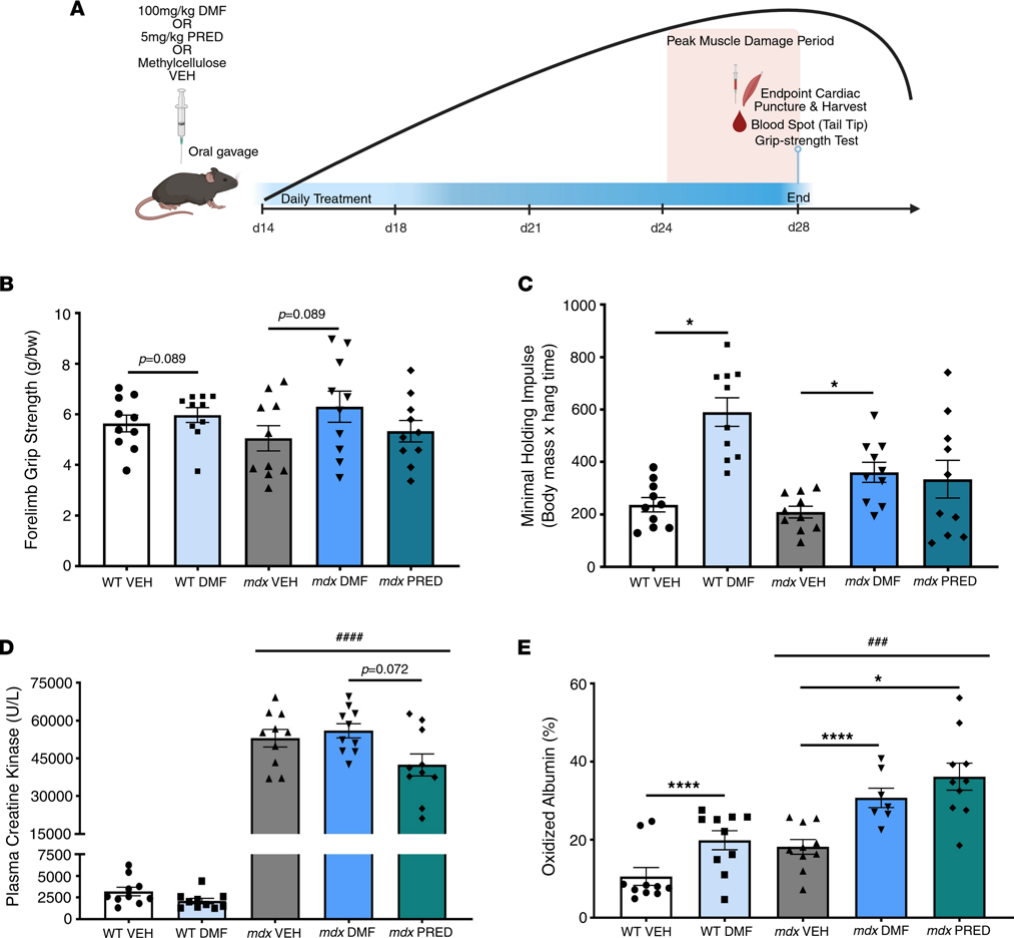
DMF improves muscle function but not DMD blood biomarkers. (**A**) Schematic of the treatment period and clinically compatible testing protocol beginning at 14 and concluding at 28 days of age. Mice were treated daily via oral gavage with vehicle (0.5% methylcellulose; VEH), 100 mg/kg DMF, or 5 mg/kg prednisone (PRED) and underwent grip strength and blood biomarker testing at the experimental endpoint at 28 days of age. (**B**) Forelimb, (**C**) whole-body grip strength, (**D**) plasma creatine kinase (CK), and (**E**) oxidized albumin levels were assessed. Data are mean ± SEM and *n* are indicated by individual data points. Statistical significance was tested by 2-way (genotype and DMF treatment) and 1-way (*mdx* treatment) ANOVA. Treatment effect: *P < 0.05, ****P < 0.0001; genotype effect: ^###^*P* < 0.001, ^####^*P* < 0.0001.

Because the *mdx* mouse manifests an overall milder DMD phenotype compared with humans, we used a juvenile period of rapid growth and muscle damage to test the tolerability and effects of short-term DMF treatment compared with standard-care PRED (and 0.5% methyl cellulose vehicle, VEH) using clinically compatible function tests and fluid biomarkers (Figure 2A). Two weeks of DMF treatment had no impact on animal welfare indices, including growth rate and food and water consumption (Supplemental Figure 1, A–D; supplemental material available online with this article; https://doi.org/10.1172/jci.insight.165974DS1), or on body weight–corrected organ mass aside from normalizing liver atrophy in *mdx* mice (Supplemental Table 1). In contrast, PRED stunted growth from day 7 of treatment (Supplemental Figure 1A; trend at day 11 *P* = 0.064), increased water consumption between 10 and 11 days (Supplemental Figure 1D), and reduced spleen mass (Supplemental Table 1).

Despite juvenile growth inducing acute severe muscle damage in *mdx* mice sufficient to raise the hematologic clinical biomarker, creatine kinase (CK), TREAT-NMD reference data report stable functional strength testing until approximately 5 weeks of age (22). Consistent with the reference, *mdx* mice showed stable forelimb (Figure 2B) and whole-body (Figure 2C) grip strength yet approximately 24-fold elevated plasma CK levels at endpoint (compared with WT; Figure 2D). Neither DMF nor PRED lowered plasma CK levels compared to *mdx* VEH (Figure 2D), yet DMF significantly increased the maximum holding impulse derived from the whole-body hang test in WT and *mdx* mice (Figure 2C). PRED had no effect on muscle function tests.

Novel blood biofluid biomarkers of DMD progression are currently being validated in patients (23) and animal models (24). Here, we assessed albumin oxidation as a biomarker of systemic oxidative stress since DMF induces the endogenous antioxidant response. Increased albumin oxidation was observed in *mdx* compared with WT blood at endpoint (Figure 2E), the theoretical “peak” of the juvenile muscle damage period. Unexpectedly, both DMF and PRED increased blood albumin oxidation (including in WT mice for DMF).

### DMF activates Nrf2 and the cytoprotective and antiinflammatory program in skeletal muscle.

**Figure 3:**
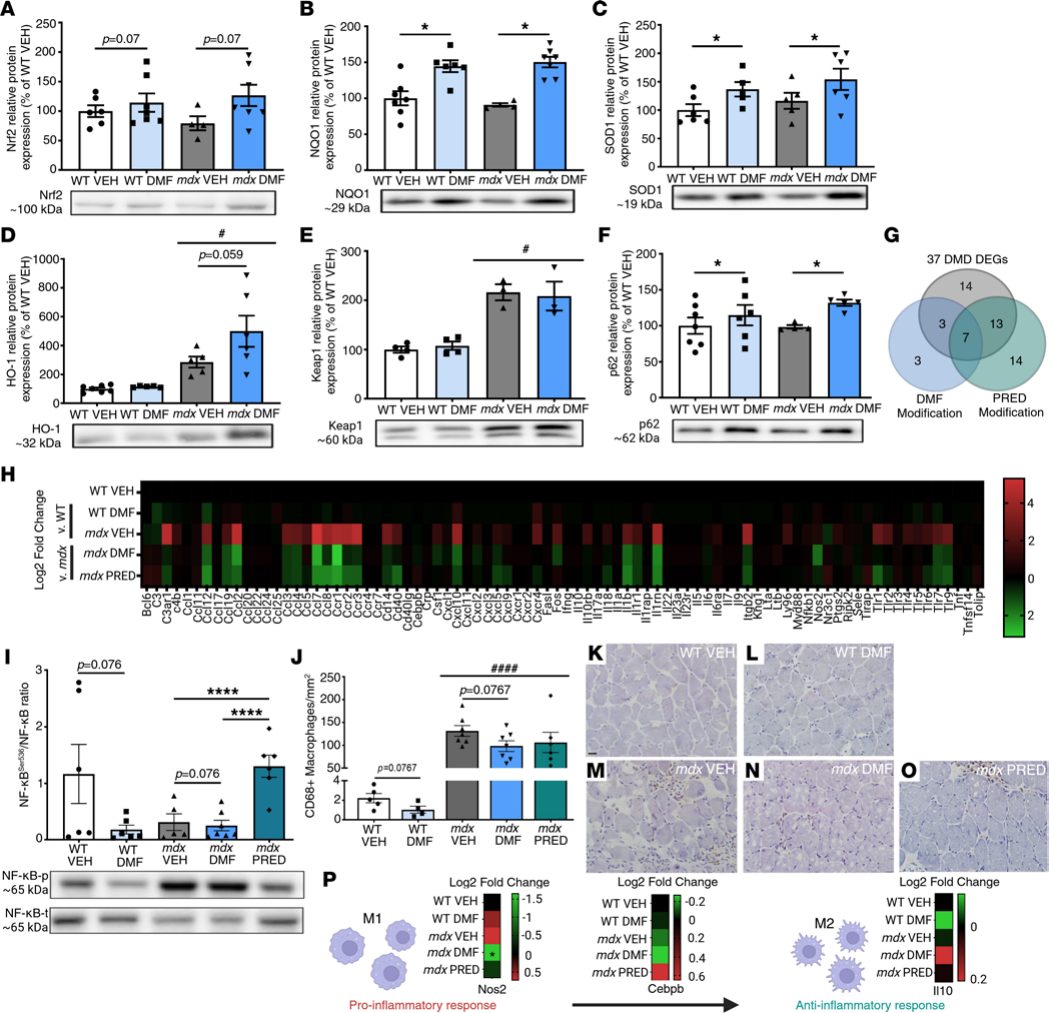
DMF activates Nrf2 and induces the phase II antioxidant response in *mdx* skeletal muscle. Protein expression of (**A**) Nrf2, (**B**) NAD(P)H dehydrogenase:quinone oxidoreductase (NQO1), (**C**) superoxide dismutase 1 (SOD1), (**D**) hemeoxygenase-1 (HO-1), (**I**) kelch-like ECH-associated protein 1 (Keap1), and (**F**) sequestosome 1 (p62) was quantitated via Western blot. (**G** and **H**) The muscle inflammatory response was assessed by quantitative real-time polymerase chain reaction (qRT-PCR) gene array. (**I**) Phosphorylated nuclear factor-κB (NF-κB) and total NF-κB protein and (**J**–**O**) CD68-positive (CD68^+^) macrophages. (**P**) Gene signatures of M1 and M2 macrophages were extrapolated from gene array data presented in **H**. Data in **G**, **H**, and **K** are based on log_2_ fold-change from WT (for *mdx* VEH) and *mdx* VEH (for *mdx* DMF and PRED) derived from *n* = 4/group where each *n* is pooled mRNA for *n* = 2 mice. Statistical significance was tested by 1-way ANOVA. **H** heatmap was partially published previously under CC BY license (73). Data presented in **A**–**F**, **I**, and **J** are mean ± SEM, and *n* are indicated by individual data points. Statistical significance was tested by 2-way (genotype and DMF treatment) and 1-way (*mdx* treatment) ANOVA. Treatment effect: *P < 0.05, ****P < 0.0001; genotype effect: ^#^*P* < 0.05, ^####^*P* < 0.0001. (**K**–**O**) Scale bar = 20 mm.

Constitutive Nrf2 synthesis outside of Keap1’s control can result in higher Nrf2 expression, although dissociation from Keap1 and translocation to the nucleus (rather than increased protein expression) is the definitive stimulus for transcription of the phase II antioxidant response. We assessed protein levels and activation of key Nrf2 regulators. Although DMF did not upregulate Nrf2 protein expression (*P* = 0.07; Figure 3A), it upregulated key phase II antioxidant enzymes including NQO1 (WT and *mdx* DMF versus VEH; Figure 3B) and SOD1 (Figure 3C). HO-1, a strong suppressor of reactive oxygen species (ROS) and inflammation, was already elevated in *mdx* muscle, and there was a trend for DMF to upregulate it further in *mdx* but not WT muscles (*P* = 0.059 *mdx* DMF versus *mdx* VEH; Figure 3D). The expression of Keap1 (Figure 3E), as well as expression of phosphorylated p62 (sequestosome 1; Supplemental Figure 2A), a classical receptor of autophagy that sequesters and tags Keap1 for degradation, were also elevated in *mdx* compared with WT muscle. DMF treatment increased p62 protein expression in WT and *mdx* muscle (Figure 3F), which maintains Keap1 binding and Nrf2 activity, but did not significantly increase p62^Ser349^ residue phosphorylation (*P* = 0.058; Supplemental Figure 2A). Neither the activity (oxidized Cys^106^), nor the protein expression of Nrf2’s molecular chaperone, DJ-1, were affected by DMF treatment, though DJ-1 protein expression was reduced in *mdx* compared with WT muscle (Supplemental Figure 2, B and C).

As well as antioxidation, DMF functions as a potent antiinflammatory and immunomodulatory drug (Figure 1). PRED is also a potent immunosuppressant to confer disease-modifying benefit in DMD. To test the immunomodulatory capacity of DMF compared with PRED, we profiled 84 inflammatory genes via qPCR RT2 gene array in gastrocnemius muscles. A total of 37 (45%) genes were differentially regulated in juvenile *mdx* compared with WT muscle (Figure 3, G and H, and Table 1). Most were associated with the acute-phase response or general regulation of inflammation. Ten DEGs were increased by more than 10-fold, which were typically chemokines or chemokine/cytokine receptors (Table 1). PRED downregulated a more extensive inflammatory gene profile than DMF (54% versus 27% of *mdx* DEGs) generally by the magnitude of 2- to 4-fold. Although modulating fewer genes, DMF downregulated the expression of key inflammatory genes by a much larger magnitude than PRED; e.g., DMF downregulated the gene expression of chemokine *Ccl7* and *Ccr1*, the type I receptor for chemokines CCL3, CCL5, CCL7, and CCL23, by 7- and 10-fold, respectively (versus nonsignificantly and 6-fold for PRED, respectively). *Ccl7* was the most DEG in *mdx* muscle (increased by 35-fold). DMF also had fewer off-target modulatory effects on normally regulated genes (NEGs) than PRED (3 versus 6 inflammatory NEGs, respectively; Figure 3G and Supplemental Table 3).

We also assessed activation and protein expression of the master regulator of innate immunity, NF-κB, which is purportedly suppressed by both Nrf2 and PRED (25). Total NF-κB protein levels were equivalent between *mdx* and WT mice, and neither DMF nor PRED treatment modulated them (Supplemental Figure 2D). Phosphorylation of the Ser^536^ activation site was also equivalent between WT and *mdx* VEH muscles (there was high variability especially in WT muscles; Supplemental Figure 2E), suggesting NF-κB signaling is crucial for muscle growth and remodeling in juvenile mice, though no more active due to dystrophin deficiency. Most surprisingly, PRED treatment increased phosphorylated (Ser^536^) NF-κB expression higher than in any other group (*mdx* PRED versus all other groups) whereas DMF tended to lower levels in both WT and *mdx* muscles (*P* = 0.058, Supplemental Figure 2E). These data were mimicked in the ratio of Ser^536^ NF-κB phosphorylation to the total NF-κB protein, a biomarker of NF-κB activity (Figure 3I).

Recruitment of immune cells to damaged myofibers is necessary for effective regeneration. Macrophages are essential in this process, driving both the inflammatory response and tissue digestion (M1, pro-inflammatory type) as well as antiinflammatory signaling essential for wound healing (M2, antiinflammatory type). The transition between M1 and M2 macrophage types is essential to prevent chronic inflammation, fibrosis, and adiposis. We assessed CD68 antigen, a pan-marker of macrophages in tibialis anterior (TA) sections. In all *mdx* groups, CD68-positive macrophages were significantly higher than WT TA sections (Figure 3, J–O). Neither DMF nor PRED significantly altered macrophage infiltrate, though (Figure 3, J and M–O). DMF but not PRED significantly reduced gene expression associated with M1 (*Nos2*, compared with VEH; Figure 3P) populations (although *Nos2* did not fall within the DEG criteria). Neither drug influenced M2 macrophage transition (e.g., *Cebpb*, *Il10*), which was normal in *mdx* compared to WT muscle (Figure 3P).

### DMF improves force production and protects against fatigue in fast-twitch muscle.

**Figure 4:**
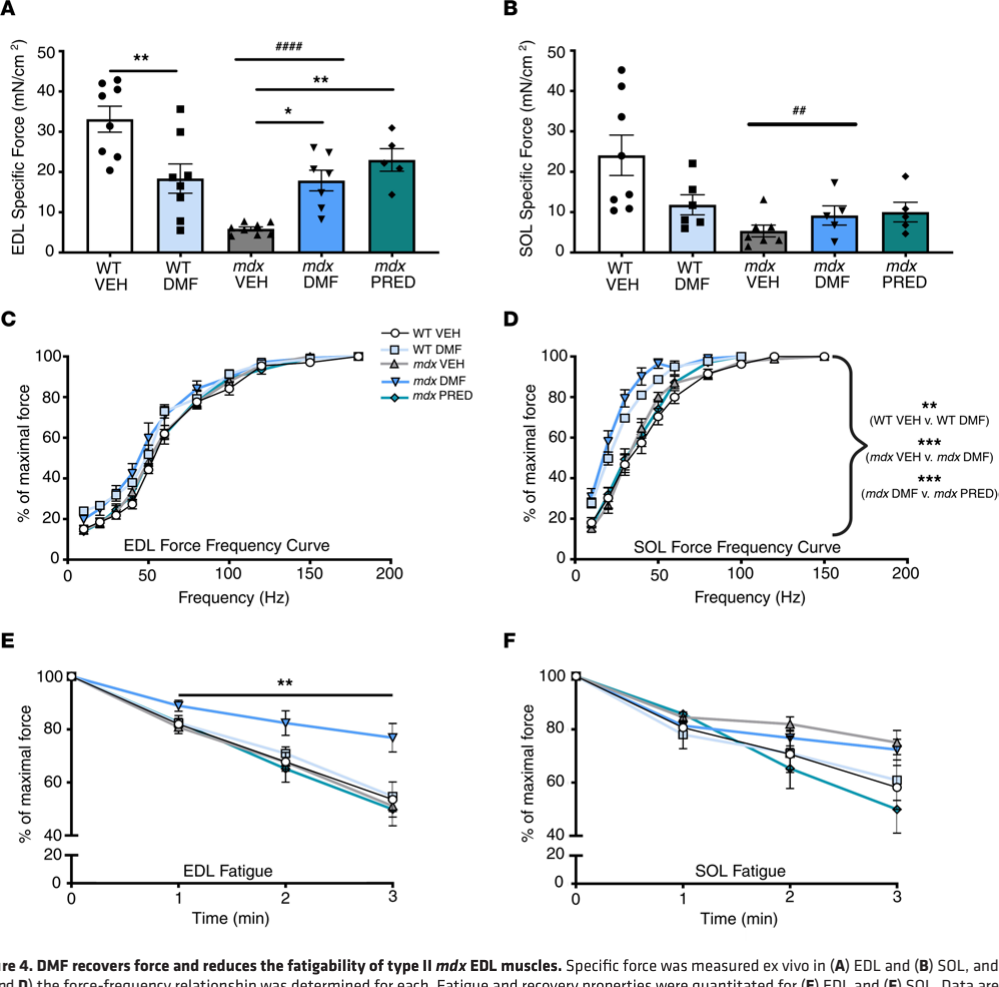
DMF recovers force and reduces the fatigability of type II *mdx* EDL muscles. Specific force was measured ex vivo in (**A**) EDL and (**B**) SOL, and (**C** and **D**) the force-frequency relationship was determined for each. Fatigue and recovery properties were quantitated for (**E**) EDL and (**F**) SOL. Data are presented as mean ± SEM and *n* are indicated by individual data points unless otherwise stated. Panel **C** *n* are WT VEH = 8, WT DMF = 8, *mdx* VEH = 11, *mdx* DMF = 8, *mdx* PRED = 7; panel **D** *n* are WT VEH = 8, WT DMF = 6, *mdx* VEH = 8, *mdx* DMF = 9, *mdx* PRED = 6; panel **E** *n* are WT VEH = 7, WT DMF = 7, *mdx* VEH = 11, *mdx* DMF = 8, *mdx* PRED = 6; panel **F** *n* are WT VEH = 7, WT DMF = 6, *mdx* VEH = 9, *mdx* DMF = 6, *mdx* PRED = 6. For data in panels **A** and **B**, statistical significance was tested by 2-way (genotype and DMF treatment) and 1-way (*mdx* treatment) ANOVA. In panels **C**–**F**, statistical significance was tested by repeated measures multivariate analysis. Treatment effect: *P < 0.05, **P < 0.01, ***P < 0.001; genotype effect: ^##^*P* < 0.01, ^####^*P* < 0.0001.

Muscle force production relative to mass is predictive of muscle quality and perhaps the most useful indicator of drug benefit on DMD progression (26). We studied ex vivo contractile characteristics in predominantly type II extensor digitorum longus (EDL) and type I soleus (SOL) muscles to scope for fiber type–specific effects of DMF and PRED (data summarized in Figure 4 and Supplemental Table 2). The specific (cross-sectional area [CSA] corrected; sPo) force was ~80% lower in EDL (*mdx* compared with WT VEH; Figure 4A) and ~60% lower in SOL (*mdx* compared with WT VEH; Figure 4B). DMF and PRED improved the sPo of EDL by >3-fold (Figure 4A) but had no significant effect on SOL (Figure 4B). DMF specifically shifted the force-frequency curve of WT and *mdx* VEH SOL, but not EDL muscles (Figure 4, C and D), indicating modulation of cross-bridge sensitivity of type I fibers. EDL and SOL muscles were subsequently subjected to a fatigue protocol, and muscle force production is shown at minute intervals (Figure 4, E and F). DMF protected *mdx* but not WT EDL muscles from fatigue (Figure 4E). SOL muscles fatigued variably across groups, but neither DMF nor PRED affected fatigability of SOL (Figure 4F).

### DMF augments mitochondrial respiration in mdx fibers through anaplerosis.

**Figure 5:**
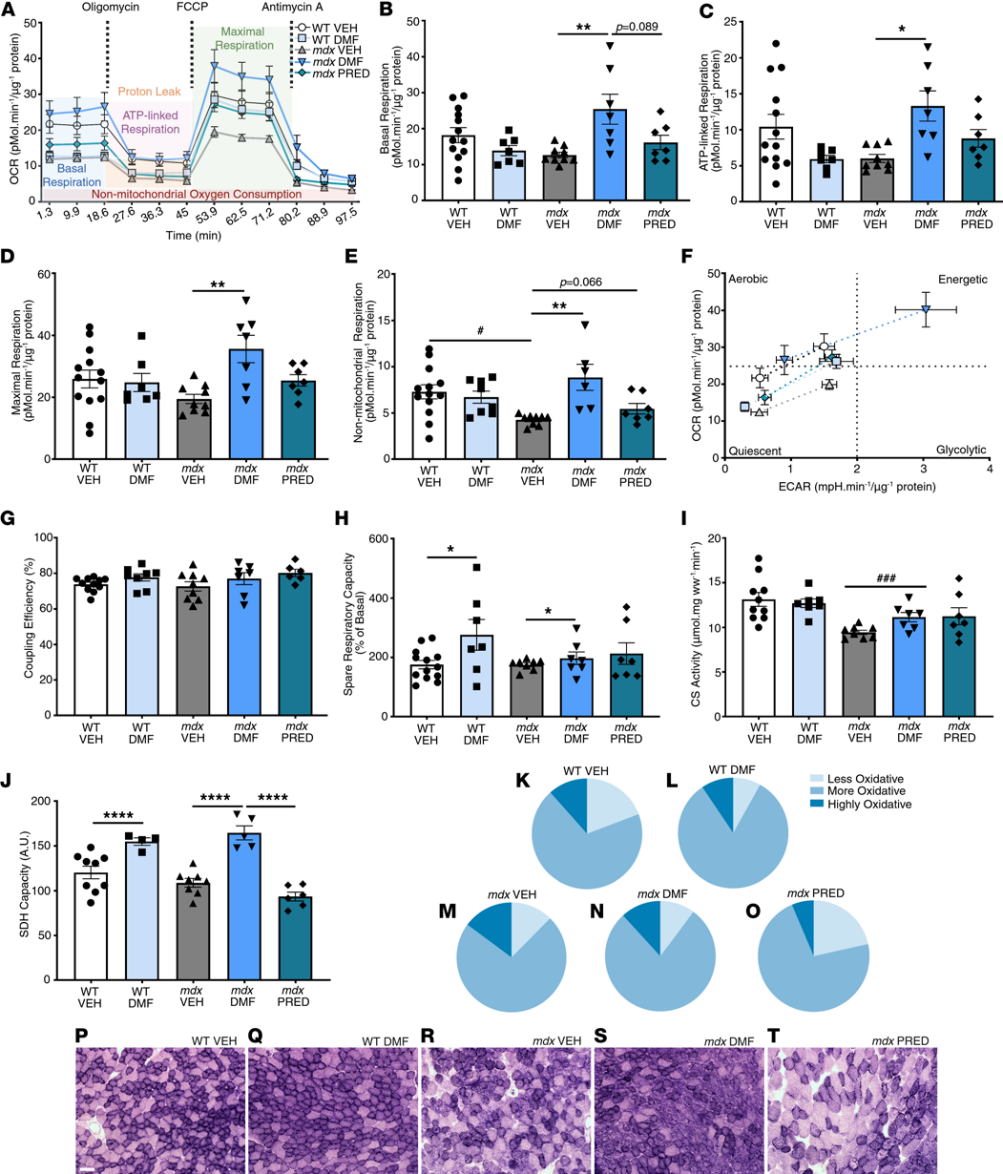
DMF enhances mitochondrial respiratory function in *mdx* FDB fibers. (**A**) Oxygen consumption rate was measured using Seahorse extracellular flux and chemical inhibitors and uncouplers of mitochondrial respiration. (**B**) Basal, (**C**) ATP-linked, (**D**) maximal, and (**E**) nonmitochondrial respiration in *mdx* fibers. (**F**) Metabolic phenotypes in response to chemical uncoupling, (**G**) coupling efficiency, (**H**) SRC, and (**I**) citrate synthase (CS) activity are also shown. (**J**) Succinate dehydrogenase (SDH) capacity was used to estimate fiber type shifts (**K**–**O**) and (**P**–**T**) representative images are shown. Data presented in **A**–**J** are mean ± SEM and *n* are indicated by individual data points unless otherwise stated. Data presented in **K**–**O** are mean percentage fiber SDH density across 3 bins. Panel **A** and **F** *n*: WT VEH = 13, WT DMF = 7, *mdx* VEH = 9, *mdx* DMF = 7, *mdx* PRED = 7. Panel **K**–**O** *n*: WT VEH = 9, WT DMF = 4, *mdx* VEH = 4, *mdx* DMF = 5, *mdx* PRED = 6. Statistical significance in **B**–**J** was tested by 2-way (genotype and DMF treatment) and 1-way (*mdx* treatment) ANOVA. Treatment effect: *P < 0.05, **P < 0.01, ****P < 0.0001; genotype effect: ^###^*P* < 0.001. (**P**–**T**) Scale bar = 50 mm.

Mitochondrial respiratory function was measured in flexor digitorum brevis (FDB) fibers using extracellular flux and a mitochondrial stress test involving the sequential application of inhibitor/stimulator drugs (Figure 5A). There was no significant difference in mitochondrial oxygen consumption in the basal (Figure 5B), phosphorylating (Figure 5C), and uncoupled states (Figure 5D). Only nonmitochondrial respiration, which is mostly attributed to cellular oxidase activity associated with antiinflammation and antioxidation, was reduced in *mdx* FDB fibers (Figure 5E). Nevertheless, DMF increased the basal, phosphorylating, maximal, and nonmitochondrial respiration (Figure 5, A–E) in *mdx* FDB fibers, resulting in an overall higher bioenergetical state (Figure 5F). There was no evidence of mitochondrial uncoupling in response to increased DMF-dependent substrate flux (Figure 5G). Spare reserve capacity (SRC) is a determinant of mitochondrial fitness/flexibility that depends on electron transport chain and inner membrane integrity, bioenergetical demand, and preservation of mitochondrial homeostasis. These factors are controlled by several signaling pathways associated with Nrf2, including cytokine-mediated STAT3 signaling (which was upregulated in *mdx* muscle but not affected by DMF; Supplemental Figure 3J), glucose and fatty acid metabolism, and oxidative stress (see ref. 27 for a review). DMF increased the SRC in WT and *mdx* muscles, consistent with Krebs cycle anaplerosis (Figure 5H).

Citrate synthase (CS) activity, a classical mitochondrial content biomarker, was reduced in *mdx* VEH gastrocnemius but was not modulated by DMF or PRED (Figure 5I), nor were protein biomarkers of biogenesis (TFAM, PGC-1α, mitochondrial complex subunits), fission (DRP-1), or fusion (OPA-1) signaling (Supplemental Figure 3, A–I). Since DMF’s (and PRED’s) protective effects on muscle force production were fiber type specific, we also assessed Complex II/succinate dehydrogenase (SDH) capacity in predominantly fast-twitch TA sections. Consistent with Krebs reversal and increased flux of malate>fumarate>succinate through Complex II, DMF increased SDH capacity in WT and *mdx* muscles (Figure 5J). Based on SDH activity staining, DMF drove a more oxidative phenotype while PRED drove a less oxidative phenotype (compared with VEH; Figure 5, K–T, and Supplemental Figure 3, K–M), demonstrating stark differences between these drugs on metabolic plasticity.

### DMF modifies biomarkers of muscle integrity, quality, and histopathology.

**Figure 6:**
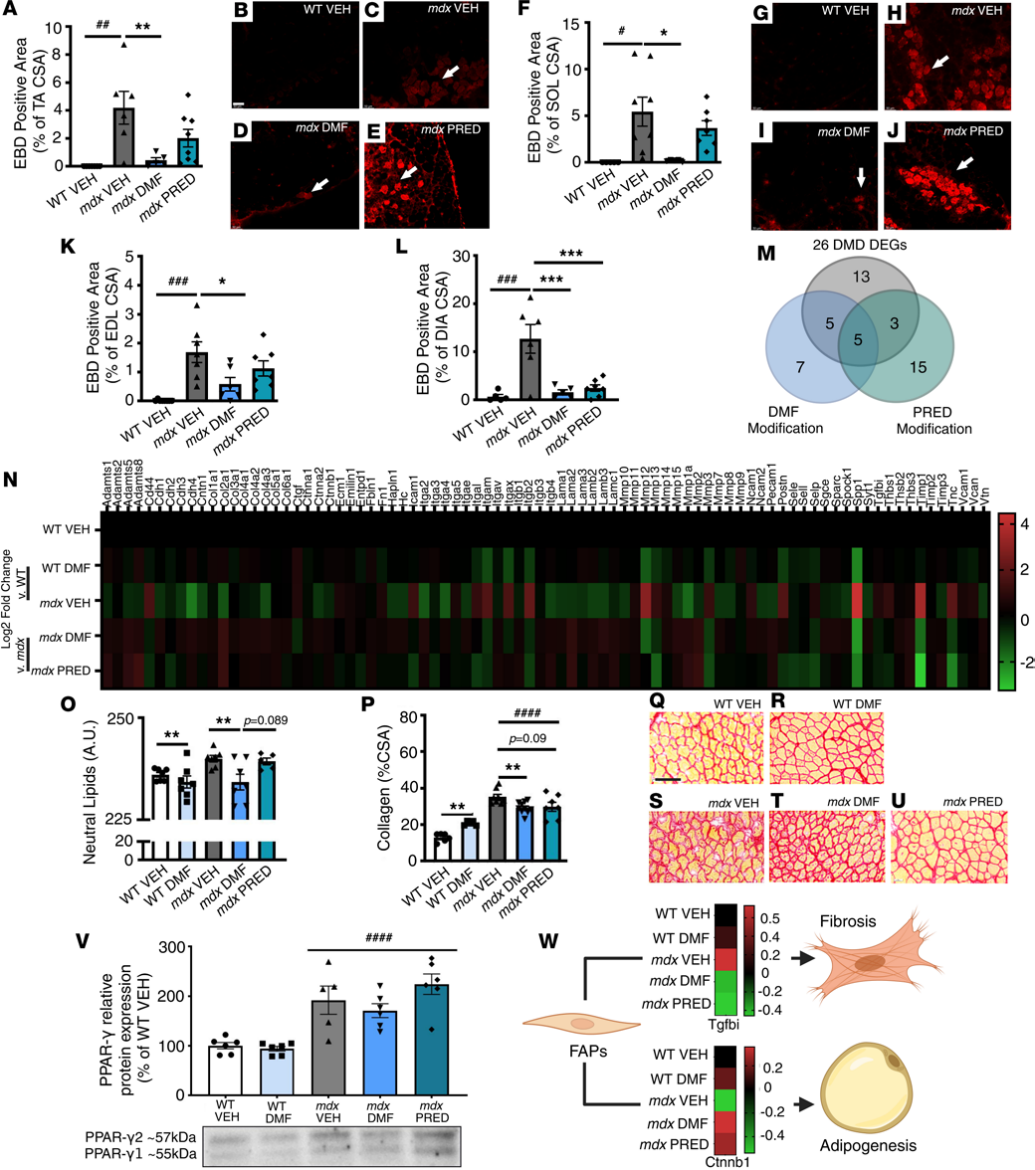
DMF improves biomarkers of muscle pathology. (**A**–**L**) EBD permeation into muscle fibers, a biomarker of compromised sarcolemma integrity, was assessed in *mdx* TA, SOL, EDL, and DIA, respectively. (**M** and **N**) An array of extracellular matrix genes were assessed alongside histological indicators of muscle (**O**) liposis and (**P**–**U**) fibrosis (collagen deposition). (**V**) Protein expression of PPARγ, an inducer of adipogenesis, and (**W**) gene expression of *Ctnnb1*, a repressor of the adipogenesis gene program and *Tgfb1*, a regulator of the fibrosis gene program, are shown. Data in **W** represent a callout from **N**. Data in **M**, **N**, and **W** are based on log_2_ fold-change from WT (for *mdx* VEH) and *mdx* VEH (for *mdx* DMF and PRED) derived from *n* = 4/group where each *n* is equivalent to pooled mRNA for *n* = 2 mice. Statistically significant dysregulated genes (panel **W**) were tested by 1-way ANOVA. For all other panels, data are mean ± SEM and *n* are indicated by individual data points. Statistical significance was tested by 2-way (genotype and DMF treatment) and 1-way (*mdx* treatment) ANOVA. Treatment effect: *P < 0.05, **P < 0.01, ***P < 0.001; genotype effect: ^#^*P* < 0.05, ^##^*P* < 0.01, ^###^*P* < 0.001, ^####^*P* < 0.0001. Panel **B**–**J** scale bar = 50 mm; panel **Q**–**U** scale bar = 20 mm.

To test whether DMF-induced Nrf2 activation could improve histopathology, a subset of juvenile WT and *mdx* mice were injected with Evans blue dye (EBD), a cell-impermeant extravasation dye that can only be absorbed by damaged muscle membranes. EDL, SOL, TA, and diaphragm (DIA) muscles were collected 24 hours after EBD injection. DMF treatment significantly reduced the percentage of EBD-positive fibers in all *mdx* muscles by up to 6-fold (Figure 6, A–L). In contrast, PRED had no effect on hind limb muscles (Figure 6, A–K) but was just as effective as DMF at reducing membrane damage of DIA muscles (Figure 6L).

In patients with DMD, muscle quality is severely compromised. As well as having less muscle due to chronic fiber degeneration, fibro-adipogenic progenitors (FAPs) within the extracellular matrix (ECM) drive reactive adiposis and/or fibrosis in response to persistent sterile inflammation signals (28). DMD muscles from patients and *mdx* mice also produce more intracellular lipid, which contributes to fatty replacement of muscle (29). We assessed early signs of fibrosis and adiposis via (i) qPCR RT2 gene array of genes controlling ECM composition and cell adhesion (Figure 6, M and N), (ii) Oil Red O (ORO) staining of neutral lipids (Figure 6O), and (iii) Picrosirius red staining of collagen (Figure 6P). A total of 26 ECM genes were differentially expressed by more than 1.5-fold (*P* < 0.05) in *mdx* compared with WT (VEH) muscle (Figure 6, M and N, and Table 2) demonstrating activation of a complex remodeling program in juvenile mice undergoing an acute disease phase. Of these DEGs, DMF modulated 10 (38%) while PRED modulated 8 (31%); 5 of the same genes were modulated by both drugs. DEGs, secreted phosphoprotein 1 (*Spp1*) and tissue inhibitor of metalloproteinase 1 (*Timp1*), were upregulated by more than 10-fold, reversibly modulated by both DMF and PRED (*Spp1* more so by DMF and *Timp1* more so by PRED; Table 2), and, in addition to *Mmp2*, defined as seed genes within the DMD disease module (30). Gene expression of macrophage elastase (*Mmp12*) was >6-fold higher in *mdx* compared with WT VEH muscle, and DMF, but not PRED, reduced expression by >2-fold. As well as modulating DEGs, PRED also downregulated the expression of 16 NEGs in *mdx* muscles (compared with 7 for DMF, Supplemental Table 3). Notably, *Mmp13*, which is crucial for muscle regeneration (31), was downregulated by 3-fold.

Muscle neutral lipid (Figure 6O) and collagen content (Figure 6, P–U) were significantly increased in *mdx* compared with WT muscle, and DMF reduced lipids in both WT and *mdx* muscles (Figure 6O) and collagen content in *mdx* muscle only (Figure 6, P–U). PRED had no effect on these indices (Figure 6, P–U). Intriguingly, DMF increased collagen production in WT muscles (Figure 6, P–R). Based on distinct cytokine signatures, FAPs proliferate and drive pro-fibrosis and adiposis programs. To determine whether the higher muscle lipid content was associated with adipogenic signaling, we assessed peroxisome proliferator–activated receptor γ (PPARγ) protein, which FAPs express upon activation of the adiposis program. PPARγ protein expression was increased in *mdx* compared with WT muscle (Figure 6V), consistent with the approximately 1.7-fold reduction in β-catenin (*Ctnnb1*) gene expression, a repressor of the FAP adipogenesis program (32) (Table 2 and Figure 6W). Neither drug affected PPARγ or *Ctnnb1* expression (Figure 6, V and W). Although fibrosis-associated gene transforming growth factor-β inducible (*Tgfbi*) just fell short of the 1.5-fold DEG classification cutoff, its expression was significantly increased in *mdx* VEH compared with WT VEH muscle (1.42-fold increase, *P* < 0.01), suggesting induction of the fibrosis program in juvenile mice (Figure 6W). DMF and PRED significantly reduced *Tgfbi* expression (both by 1.6-fold, Figure 6W).

**Figure 7:**
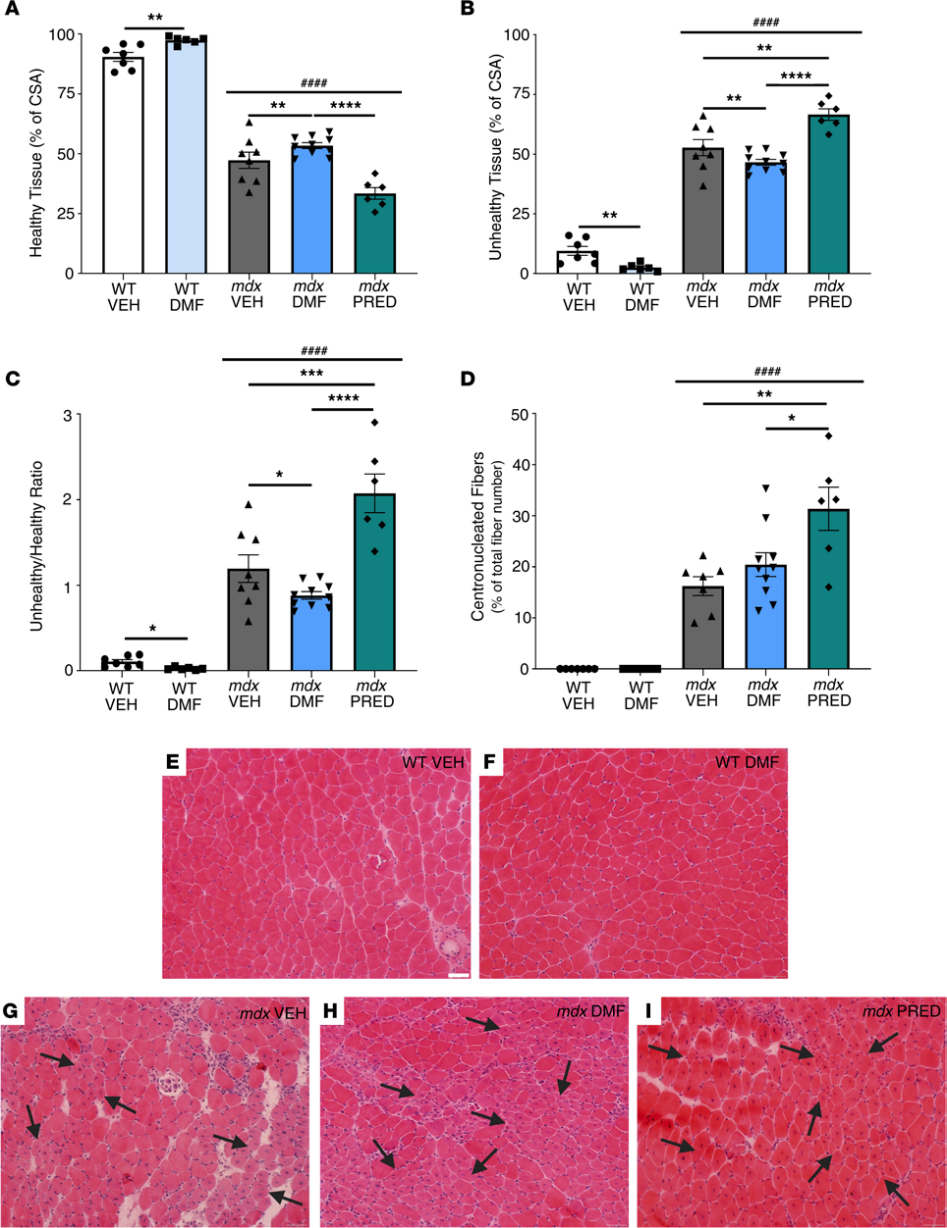
DMF improves *mdx* muscle histopathology. TA architecture was assessed using hematoxylin and eosin staining. The (**A**) healthy and (**B**) unhealthy tissue as well as (**C**) the unhealthy to healthy tissue ratio and (**D**) percentage regenerating centronucleated fibers are shown. Representative images of (**E**) WT VEH, (**F**) WT DMF, (**G**) *mdx* VEH, (**H**) *mdx* DMF, and (**I**) *mdx* PRED TA muscles are provided where arrow pointers indicate regenerating centronucleated fibers. Statistical significance was tested by 2-way (genotype and DMF treatment) and 1-way (*mdx* treatment) ANOVA. Treatment effect: *P < 0.05, **P < 0.01, ***P < 0.001, ****P < 0.0001; genotype effect: ^####^*P* < 0.0001. (**E**–**I**) Scale bar = 50 mm.

Finally, we sought to understand whether DMF could temper muscle degeneration through Nrf2-mediated cytoprotection. Classical indicators of muscle histopathology were assessed in TA sections using H&E staining to derive the proportion of healthy (intact, peripherally nucleated fibers) and unhealthy (regenerating centronucleated fibers, degenerating fibers, and inflammatory infiltrate) muscle (Figure 7). Healthy muscle was reduced, unhealthy muscle was increased, and the unhealthy/healthy tissue ratio was higher in *mdx* than WT muscles (Figure 7, A–C, E, and G). DMF increased the healthy muscle proportion (Figure 7A) and reduced the unhealthy muscle proportion in WT and *mdx* mice, resulting in reduction of the unhealthy/healthy tissue ratio (Figure 7, A–C and E–H). In contrast, PRED increased the proportion of unhealthy muscle (Figure 7B), specifically the relative area of centronucleated regenerating fibers, compared with both *mdx* VEH and *mdx* DMF TAs (Figure 7, D, G, and I). There was no evidence of global muscle atrophy due to genotype or treatment, although there was some redistribution of fiber size due to genotype (more smaller fibers in *mdx* groups) and PRED treatment (more medium-sized fibers; see Supplemental Figure 4, A–G).

## Discussion

DMD is a difficult disease to clinically manage because of its pathological complexity. Glucocorticoids have persisted as standard pharmacological care because despite their side effect profile, there is no better alternative. Experimental therapeutics targeting many aspects of DMD pathobiology are in the clinical pipeline (reviewed in ref. 15). However, translational success rates have been low, highlighting that a different approach to investigational drug selection is required. In this study, repurposed DMF was investigated for potential therapeutic benefit relative to standard-care PRED due to its multimodal effects (reviewed in ref. 18). To our knowledge, this is the first study to investigate DMF for myogenic disease, and our molecular data indicate dystrophic skeletal muscle uptake and pharmacodynamic action (e.g., 50% increase in NQO1, unchanged HO-1) as biomarked previously in the context of rheumatoid arthritis (30% increase in NQO1, unchanged HO-1) and RRMS (15% increase in NQO1, unchanged HO-1) (12). We showed that both DMF and PRED improved the force output of EDL (composed predominantly of type II myofibers and the least damaged muscle as shown by EBD fluorescence), protected against sarcolemma damage (i.e., EBD uptake) of DIA muscles, and modified an extensive list of inflammatory and ECM-modulatory genes. However, only DMF consistently reduced sarcolemma damage and histopathology of hind limb muscles and increased performance on a clinically compatible function test. Nrf2 activator compounds, sulforaphane and curcumin, also reduce EBD fluorescence in *mdx* muscle fibers (21) while DMF specifically tempers dysregulated phospho- and sphingolipid metabolism by inhibiting damaging lipases (33), highlighting membrane protection mediated via Nrf2. The mechanisms likely involve enhanced antioxidant defenses and/or membrane stabilization of basal lamina and ECM components. DMF increased protein levels of key antioxidative enzymes (NQO1 and SOD1; Figure 3) and normalized genes involved in basal lamina composition (e.g., various *Mmp*s; Table 2) and the control of ECM environments (e.g., *Sparc*: ↓1.69-fold in *mdx* vs. WT, ↑1.27-fold by DMF, *P* < 0.05; Table 2). In contrast, PRED conferred no protection on sarcolemma membranes of hind limb muscles and appeared to slow regeneration (e.g., more centronucleated fibers, 3-fold reduction in myogenesis regulator *Mmp13*, and increased NF-κB signaling) during the acute severe disease phase in juvenile mice. Effective muscle regeneration depends on coordinated immune and transitional ECM signaling. Centronucleation can persist in regenerating/repairing myofibers for variable durations (up 94 weeks following chemotoxic muscle injury) and is associated with both satellite cell–mediated and myofiber-autonomous repair mechanisms (34). The potent pan-immunosuppression conferred by PRED appears too strong to support expedient repair as recently indicated in juvenile *mdx* mice. This may be a mechanism through which PRED constrains muscle size in DMD in addition to its atrophic effects mediated through antagonism of the insulin receptor (35).

DMF was particularly effective against histopathological hallmarks of myopathy, including sarcolemmal damage, muscle degeneration, inflammatory (particularly macrophage) infiltrate, liposis, and collagen deposition. Difficult to resolve in the context of our positive histopathology data, though, is that DMF could not abrogate clinically relevant hematologic biomarkers of DMD (e.g., CK or oxidized albumin) or improve force output of SOL despite conferring significant sarcolemma protection to this muscle (Figure 6, F–J). Rather both DMF and PRED drove systemic hematologic oxidation, which may be crucial to their immunomodulatory MOAs. Previous studies in patients with RRMS showed DMF transiently elevates the oxidative state of peripheral blood by driving ROS production in monocytes (36). This “oxidative burst” appears crucial for DMF’s immunomodulatory precision. To our knowledge, these data are the first to demonstrate that PRED acts in a similar fashion. Induction of the endogenous antioxidant response in muscle, particularly in highly oxidative fibers, which almost exclusively compose mouse SOL and have a higher innate antioxidant capacity because of their mitochondrial density, may come at the expense of force output via oversequestration of ROS. In physiological ranges, ROS are essential for modulating cross-bridge cycling kinetics to increase force production, though at supraphysiological levels, they dose-dependently reduce force output (reviewed in ref. 37). Longitudinal testing is required to determine whether DMF can alter systemic muscle wasting across all fiber types sufficiently to modify disease course as indicated by plasma CK levels. However, we note that PRED does not affect plasma CK levels in *mdx* mice (38–40) or sometimes in DMD patients (41) despite improving muscle force/function indices. The same may be true for DMF, highlighting that histopathological modification is a more definitive indicator of disease-modifying capability.

Disease and treatment omics data are useful tools to map pathobiologic pathways, identify disease drivers and potential drug targets, and enable efficient drug selection, especially for complex diseases like DMD (1). Recently, a 5 seed gene–driven interactome was identified through computational meta-analysis of studies quantitating muscle gene expression in patients with DMD: drugs developed for multiple sclerosis (MS), other autoimmune diseases, and hematological cancers were revealed as ideal repurposing candidates (30). DMF has proven efficacy in the clinical treatment of RRMS and the autoimmune disease psoriasis. It is also currently being investigated for the treatment of acute myeloid leukemia (8). Our quantitative real-time polymerase chain reaction (qRT-PCR) gene array studies captured 4/5 of Lombardo et al.’s (30) disease module seed genes (Figure 6, Table 2, and Supplemental Table 3), namely in order of hierarchy in the interactome: *Mmp2*, *Spp1*, *Timp1*, and fibronectin 1 (*Fn1*). DMF effectively lowered the expression of all 3 fibrosis-associated DEGs (*Mmp2*, *Spp1*, and *Timp1*) but did not affect *Fn1* (Figure 6, Table 2, and Supplemental Table 3). Conversely, PRED significantly downregulated *Fn1*, whose upregulation is critical for augmenting muscle repair mechanisms (42). As a point of contrast, PRED better modulated expression of *Timp1*, which is induced by cytokines and whose protein product inhibits MMP-mediated collagen degradation, while DMF was more effective against *Spp1*/osteopontin (OPN) and *Mmp*s (Figure 6F and Table 2). This finding is important because OPN ablation lessens severity of the *mdx* phenotype by skewing macrophage polarization to a pro-regenerative over a pro-fibrogenic phenotype (43). *Spp1* genotype and overexpression are also associated with a rapidly progressive DMD phenotype and are therefore utilized as a predictive biomarker of disease course (44). *Ccl7* was the most DEG in *mdx* muscle; its protein product interacts with MMP2 via CCR2 (45). Dysregulated CCL7 is implicated in, and worsens, immunological diseases, including MS (46) and psoriasis (47), against which DMF is particularly effective. In addition to *Mmp2* gene expression (normalized by DMF and PRED), DMF (but not PRED) reduced the expression of macrophage-activated degrader of ECM elastic elements, *Mmp12*, by half. This could explain how DMF normalized the longer optimum length observed in *mdx* compared with WT EDL and SOL muscles during our contractile studies (Supplemental Table 2), although the significance of this finding is unknown since DMF improved contractile function exclusively in *mdx* EDL. While we did not capture insulin-like growth factor 1 (*Igf-1*) transcription in this study, others have demonstrated (i) IGF-1 levels decrease as DMD progresses, and (ii) DMF and analogous fumarate esters increase *IGF-1* expression in neurons (48), while glucocorticoids notoriously reduce circulating IGF-1 levels (49). Collectively, these data suggest DMF could modulate a selective disease gene network over eliciting pan-immunosuppression like PRED appears to, at least at the singular doses examined in this study. Although corticosteroids are useful to abate the immune system during the initial phase of an MS relapse, only DMF can modulate the immune system over the long term to reduce relapse rate (50).

A distinct effect of DMF over PRED treatment was on mitochondrial function. Our data show acute DMF treatment augments mitochondrial respiration through increased substrate flux rather than via mitochondrial homeostasis signaling. Channeling fumarate through the malate-aspartate shuttle into the mitochondrial Krebs cycle can reverse flux, driving mitochondrial respiration through Complex II/SDH, and is driven endogenously by the purine nucleotide cycle during metabolic stress (29). Although we saw no evidence of altered mitochondrial function in juvenile *mdx* FDB fibers, mitochondrial anomalies are well reported in animal models of DMD and patients, including in muscle stem cells (reviewed in refs. 29, 51). Complex I dysfunction has been reported in isolated mitochondria (52) and fibers (53) from *mdx* mouse muscle. ATP production/phosphorylating respiration can be partially restored by rerouting respiration through Complex II (via addition of succinate and Complex I inhibitors; ref. 52). Our data suggest that DMF can achieve a similar mechanism during its end stage metabolism within the Krebs cycle. This mechanism likely confers fatigue resistance in response to repetitive contraction as shown in *mdx* muscles in our contractile studies. Nrf2 activation can also augment purine nucleotide biosynthesis to enhance bioenergetics (54). Impaired mitochondrial homeostasis mechanisms, e.g., mitochondrial biogenesis and fission-fusion dynamics, are also described in dystrophin-deficient muscles and can be rescued via Nrf2 activation, although we saw no evidence of changes to crude protein markers with acute DMF treatment. Several mitochondrial targeted therapeutics (e.g., ^(*+*)^epicatechin, MA-0211, elamipretide) are currently in clinical trials in patients with DMD, but none have shown efficacy in slowing disease course yet (55). In fact, a phase IV idebenone trial was discontinued in 2020 due to futility (56), indicating that exclusively targeting mitochondria may be insufficient to slow the clinical course of DMD. DMF could represent a better alternative because it can modulate multiple drivers of DMD pathobiology, including at the mitochondrial level.

Ultimately, the benefit of a drug to a patient population weighs efficacy against the unwanted side effect profile. PRED’s profile is extensive, restricting therapeutic application to all patients despite its disease-modifying benefits, especially to young patients and over the long term. We showed adverse effects on growth, fluid intake, and spleen size in mice after only 2 weeks of PRED treatment, consistent with other studies (57–59). We commenced treatment prior to the onset of the acute severe MD phase at ~18 days (i.e., treatment began at 14 days of age) to give maximum chance for therapeutic efficacy and attenuation of severe phasic DMD. Early treatment of patients with DMD with glucocorticoids is recommended for the same reason (60). Ambulatory DMD patients treated with PRED show shorter stature, heavier weight, and greater body mass index compared with steroid-naive patients, and earlier commencement, higher dosage, and longer duration are predictive of growth retardation (61). Our data are consistent with the known growth-inhibiting, mineralocorticoid, and immunosuppressive side effects of PRED treatment in children (62). In contrast, DMF had no impact on growth or on the mass of any organ assessed in our mice, except for normalizing *mdx*-specific liver atrophy. While it does have known side effects in humans — namely, flushing, gastrointestinal disturbances, and, in rare cases, leukopenia — we saw no adverse impact of DMF treatment on animal welfare parameters. Most of DMF’s side effects can be prevented or alleviated through dose ramping and timing intake with food, and more recently developed fumarate ester drugs, such as diroximel fumarate, have far fewer side effects (8). DMF was shown to be safe and efficacious in a 13-month multicenter study in pediatric MS patients with no impact on growth (63), highlighting that if it were to impart PRED-equivalent efficacy against DMD over the long term, it could prove a superior drug based upon side effect profile alone.

In summary, the data highlight acute DMF treatment as a robust modulator of the DMD disease module leading to extensive histopathological and functional benefits over PRED treatment. Follow-up preclinical studies are required to understand whether DMF can slow the progression of murine DMD especially over the long term. These studies should include multidose comparisons of DMF alone and in combination with lower dose PRED, since notable limitations of our study were that (i) only a singular DMF dose was investigated; (ii) our selected dose of PRED was on the higher end and was delivered daily rather than intermittently, which was more recently shown to elicit human-comparable efficacy (35, 64); and (iii) additive treatment with PRED was not assessed. Additive effects are important to determine because clinical trials will inevitably involve patients receiving glucocorticoid standard care. Future studies could also pre-empt the replacement of PRED over the next decade with new-wave synthetic corticoids that boast fewer side effects and compare DMF alongside and additive to, for example, valmorolone (65).

## Methods

### Animals

#### Breeding, housing, and care.

Dystrophin-positive C57BL/10ScSn WT mice and dystrophin-negative C57BL/10 *mdx* (*mdx*) mice were bred from stock originally sourced from Animal Resources Centre (Western Australia, Australia) at the Western Centre for Health, Research and Education Animal Facility (Sunshine Hospital, Victoria, Australia), on a 12-hour light/12-hour dark cycle, 20°C–25°C, 40% humidity. Animal welfare was monitored daily to accurately determine litter birth dates. Once born, litters remained in cages until weaning age (21 days). Thereafter, litters were housed in cages of 3–10 in treatment groups for the remainder of the study. From this point, food and water consumption and body weight were monitored daily. Dystrophin deficiency and dystrophin-complexed protein downregulation in *mdx* mice were confirmed via Western blot (Supplemental Figure 5).

#### Treatment protocol.

Homozygous littermates (male and female) were randomly assigned to treatment groups at 14 days of age. Our preliminary data indicate WT and *mdx* juvenile male and female mice performed comparably on preclinical functional and blood biomarker (CK) testing (Supplemental Figure 6). WT and *mdx* mice were treated with either 0.5% methyl cellulose VEH (v/w) or ground DMF suspended in 0.5% methyl cellulose (v/w). A third cohort of *mdx* mice were treated with PRED suspended in 0.5% methyl cellulose (v/w). Animals were weighed daily (in the morning), and individual treatments were prepared relative to body weight to give a final daily dosage of either 100 mg/kg/d DMF or 5 mg/kg/d PRED. These dosages are consistent with previous preclinical studies of DMF for MS (8) and preclinical studies evaluating other drugs against *mdx* MD compared with PRED (66). Treatments were administered via oral gavage using a 21G gavage needle, and animals were monitored for adverse events for ~5 minutes postgavage. Animals were treated up to and at 27 days of age (i.e., 14 days of treatment).

#### Functional muscle strength testing.

At 28 days, forelimb grip strength was measured using a commercial dynamometer (Bioseb) over 3 consecutive efforts with 30 seconds of rest in between. The maximal effort (g) was used as absolute force (g) and was corrected for body mass (g/g). After 5 minutes of rest, mice were subjected to a 4-limb hang test using a grid mesh system (custom) to assess whole-body strength. Mice were excluded if they refused the test (hanging < 10 seconds on 3 repeated attempts). The minimal holding impulse was calculated as body mass multiplied by absolute hang time. Experimenters were masked to group assignment for muscle function testing.

#### Blood biomarkers.

After functional tests were performed on day 28, tail ends were snipped, and blood was collected onto a PerkinElmer 226 Spot Saver RUO Card containing polyethylene glycol maleimide. Cards were stored with silica gel desiccant for transport to the University of Western Australia. Albumin was extracted into 0.05% Tween 20 in 20 mM phosphate with further binding to Cibacron Blue F3GA agarose, then eluted with 25 μL of 1.4 M NaCl in 20 mM phosphate buffer pH 7.4. Gel electrophoresis, imaging, and calculation of total albumin oxidation were performed as Lim et al. described (67). On day 28, mice were anesthetized (4% induction, 2.5% maintenance isoflurane), and blood was collected via terminal cardiac puncture into lithium heparin microtubes. Plasma was derived by centrifugation (3,000*g*, 5 minutes, 4°C), and CK was quantitated spectrophotometrically using a commercially available kit (Randox Laboratories).

#### EBD treatment.

A separate cohort of (male and female) mice was utilized for EBD detection of skeletal muscle damage. This is because EBD interferes with standard histological staining protocols and fluorescence-based assays (such as extracellular flux) and may affect physiological parameters. Mice were injected with 1% EBD in saline (at 1% v/w) on day 27, exactly 24 hours prior to tissue harvest and following the final gavage treatment.

#### Surgical procedures.

On day 28, animals were weighed, deeply anesthetized (4% induction and 2.5% maintenance isoflurane), and used for ex vivo experiments. Hind limb skeletal muscles were surgically excised in the following order: FDB, EDL, SOL, TA, plantaris, gastrocnemius, and quadriceps. Organs/muscles were removed in the following order: DIA, heart, lungs, liver, spleen, and kidneys. Muscles and organs were weighed, then processed for experiments.

### Metabolic studies

#### Mitochondrial respiration and extracellular acidification.

Isolated FDB fibers were prepared from whole FDB muscles as we described previously (68) with modification to incubation time (50 minutes instead of 1.5 hours). Mitochondrial oxygen consumption and extracellular acidification rates were measured using a standard mitochondrial stress test on a Seahorse extracellular flux analyzer (Agilent).

#### CS activity.

CS is the first enzyme of the Krebs cycle and an accepted biomarker of mitochondrial density. CS activity was determined as we described previously (68).

### Ex vivo muscle contractile function studies

Ex vivo muscle contractile properties was performed masked as described by us previously on EDL and SOL using Danish Myo Technology (69). Data were excluded where muscles were indicated to be damaged by dissection or overstretching based on masked post hoc tetanic force curve analysis.

### Muscle histopathology

From EBD-treated mice, TA, EDL, SOL, and DIA strips were coated in OCT (TissueTek) and snap-frozen in chilled 2-methylbutane (in LN_2_; MilliporeSigma), and only TA was collected from all other non–EBD-treated mice. Processed muscles were serially cryosectioned (10 μm at –15°C).

EBD sections were fixed in acetone (–15°C) and mounted with DPx (BDH). Slides were imaged using TRITC-filtered fluorescence microscopy at ×40 original magnification (BX53 Olympus Fluorescence Microscope). EBD-positive fibers were quantitated using ImageJ (NIH) and expressed relative to the muscle CSA.

TA cryosections from non–EBD-treated mice were stained with a standard H&E protocol (68). To generate fiber size frequency distributions, all fibers on the cross section were individually traced on a Microsoft Surface tablet using ImageJ. For the quantitation of healthy versus nonhealthy tissue, peripherally nucleated myofibers were distinguished from centronucleated myofibers, and each group were counted and expressed relative to the total fiber number in the cross section using ImageJ as previously described (70). Degenerating tissue was quantified as previously described (68).

SDH (Complex II) activity/capacity was also quantified in TA sections as described previously (68). Using ImageJ, images were deconvoluted (Haematoxylin and Periodic Acid of Schiff’s vector), and SDH activity–positive intensity density was quantified on the purple split relative to the total CSA.

Neutral lipid droplets were quantified in TA sections as described previously (68). Using ImageJ, images were deconvoluted (Fast Red: Fast Blue vector), and ORO-positive intensity density was quantified on the red split relative to the total CSA.

Picrosirius red staining was used to quantify muscle collagen (type I and III) deposition. Eight-bit images were thresholded and percentage collagen-positive area was derived via unbiased automated quantitation using ImageJ.

Macrophage infiltration was quantified as performed previously (71). TA cryosections of 10 μm were fixed in 4% paraformaldehyde, then reacted with an anti-CD68 primary antibody, a pan macrophage marker (Abcam; ab125212; 1:500 dilution; 90 minutes’ incubation), followed by incubation with an anti-rabbit HRP-linked secondary antibody (Vector Biolabs; PI-1000-1; 1:750 dilution; 75 minutes’ incubation). The DAB substrate chromogen was used to visualize CD68-positive cells. Nuclei were counterstained with Harris hematoxylin. CD68-positive cells were manually counted using ImageJ and expressed as number of cells per square millimeter of muscle cross section.

Unless otherwise stated, slides were imaged on a Zeiss Axio Imager Z2 scanning microscope at ×200 original magnification. The experimenters were masked to group assignment for all histopathological analyses.

### Protein expression

Western blotting was used to determine target engagement of Nrf2 by DMF and downstream cell signaling of the antioxidant and antiinflammatory responses, mitochondrial dynamics, cell stress, as well as cytoskeletal proteins, in gastrocnemius as described by us previously (72). Primary antibodies used were anti–DJ-1 and anti–DJ-1 Cys^106^ (from Craig Goodman, Centre for Muscle Research, University of Melbourne), anti-Desmin (1:1,000; 5332; Cell Signaling Technology [CST]), anti-DJ1 (1:1,000; 5933; CST), anti-Dystrobrevin (1:500; 610766; BD Biosciences), anti-Dystrophin (1:500; ab15277; Abcam), anti–DRP-1 (1:1,000; 8570; CST), anti–HO-1 (1:1,000; ADI-SPA-896; Enzo Life Sciences), anti-KEAP1 (1:1,000; 8047; CST), anti–NF-κB (1:500; 8242; CST), anti–phospho–NF-κB (1:500; 3033; CST), anti-NQO1 (1:1,000; 62262; CST), anti-Nrf2 (1:1,000; 12721; CST), anti–OPA-1 (1:1,000; 80471; CST), anti–phospho-p38 (1:750; 4511; CST), anti-p62 (1:1,000; 5114; CST), anti–phospho-p62 (1:500; 95697; CST), anti–PGC-1α (1:1,000; AB3242; MilliporeSigma), anti-PPARγ (1:1,000; 2492; New England Biolabs), anti–SOD-1 (1:3,000; ADI-SOD-101; Enzo Life Sciences), anti-STAT3 (1:1,000; 12640; CST), anti–phospho-STAT3 (1:2,000; 9145; CST), anti-TFAM (1:1,000; ab252432; Abcam), and total OXPHOS cocktail (1:1,000; ab110413; Abcam). Membranes were probed with an HRP-conjugated secondary antibody (1:5,000; anti-rabbit IgG or 1:20,000; anti-mouse IgG, Vector Laboratories) in 5% nonfat milk powder in TBS-Tween (1 hour, room temperature), then stained with Coomassie blue and normalized to total protein.

### Gene arrays

Mature messenger RNA was isolated from quadriceps homogenates (RNeasy Mini Kit, QIAGEN). Cell lysates were transferred onto RNeasy mini-spin columns, and DNA was removed using DNase digestion/treatment (RNase-Free DNase Set, QIAGEN). The RNA integrity number (RIN) was quantified for all samples (Agilent RNA 6000 nano kit and 2100 Bioanalyzer), and RIN values above 7.5 were used as the inclusion criterion for subsequent gene expression analysis. RNA concentration was subsequently measured (Qubit RNA BR Assay, Invitrogen) in triplicate, and aliquots of each sample were reverse-transcribed to make cDNA (RT2 first strand kit, QIAGEN). qRT-PCR was performed using Mouse Inflammatory Response and Autoimmunity (PAMM-077Z) and Mouse Extracellular Matrix and Adhesion Molecules (PAMM-013Z) RT2 Profiler PCR arrays (QIAGEN). CT values were normalized based on a selection of reference genes (*ACTB*, *B2M*, *GAPDH*, *GUB*, *HSP90AB1*) and fold-changes/regulation of gene expression were calculated using the 2^(-ΔΔCT)^ formula (GeneGlobe, QIAGEN). Differential expression of genes (up- and downregulation) was identified using the criteria of a >1.5-fold increase/decrease in gene expression and *P* < 0.05 from the reference group. Heatmaps were created using log_2_-transformed *z* scores.

### Statistics

Data are reported as mean ± SEM unless otherwise stated. Two-way ANOVA was used for all analyses with genotype and DMF treatment as factors. One-way ANOVA was used to assess PRED treatment relative to other groups. Repeated measures analysis was used for body weight, food and water consumption, grip strength, muscle force frequency, and fatigue studies. Tukey’s post hoc test was used for multiple comparisons. A *P* < 0.05 was considered significant and trends were reported at *P* < 0.1. DEG criteria were >1.5-fold regulation and *P* < 0.05. The NEG criterion was <1.5-fold regulation. Data points were considered outliers and removed from the set if they fell ±2 standard deviations from the mean. For ex vivo contractile studies, muscles can be inconspicuously damaged during surgical excision. Muscles that produced an irregular tetanic force response were excluded from further analysis.

### Study approval

Mice used in this study were generated from a breeding program approved by the Victoria University Animal Ethics Committee (AEETH 17-010, superseded by AEETH 20-005). At 14 days of age, mice were transferred to an approved experimental project (AEETH 17-007, superseded by 19-003). Animals were bred and cared for according to the Australian Code of Practice for the Care and Use of Animals for Scientific Purposes guidelines.

### Data availability

All individual data values represented in graphs (main and supplemental) are available in the Supporting Data Values file.

## Author contributions

ER, CAT, and SK drafted the manuscript. ER, CAT, DF, and NG conceived the study. CAT, ER, SK, DAD, DGC, ND, and APB conducted experiments and analysis. NP, EJR, RMB, DD, and LS conducted experiments. ER, DF, CAT, JRT, PGA, VA, NS, and JBDH provided technical know-how and resources and interpreted data. PH provided intellectual input on the manuscript. All authors reviewed the manuscript. Order of co–first authors is assigned alphabetically (first name).

## Supplementary Material

Supplemental data

Supporting data values

## Figures and Tables

**Table 1:**
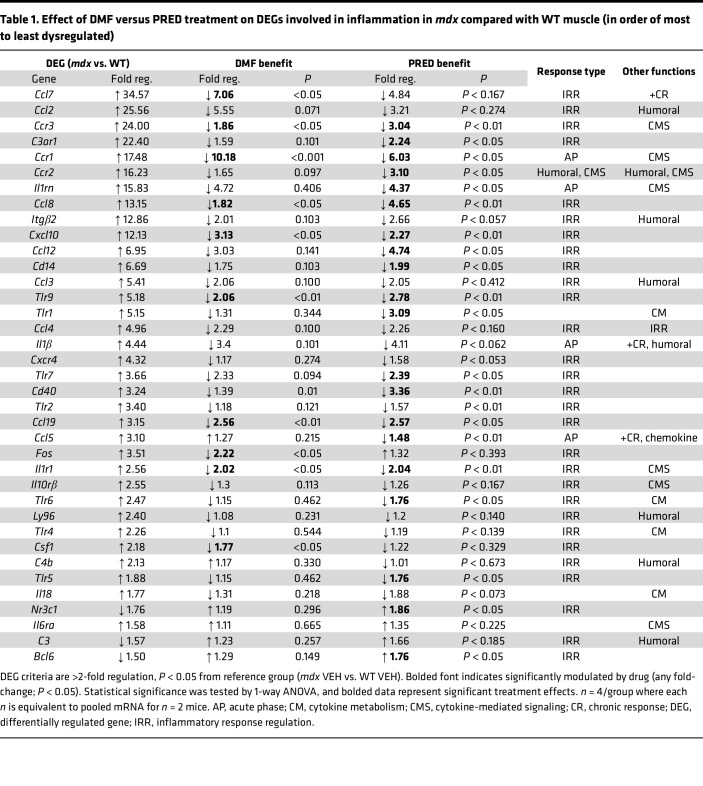
Effect of DMF versus PRED treatment on DEGs involved in inflammation in *mdx* compared with WT muscle (in order of most to least dysregulated)

**Table 2:**
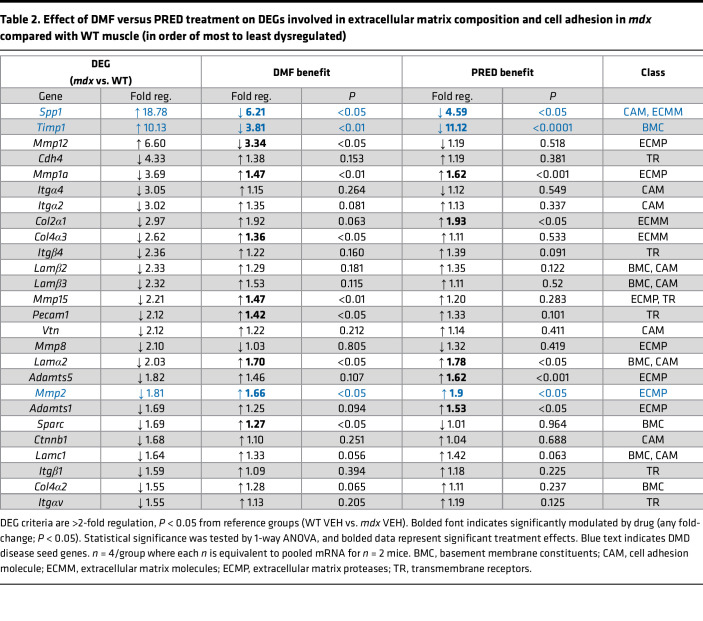
Effect of DMF versus PRED treatment on DEGs involved in extracellular matrix composition and cell adhesion in *mdx* compared with WT muscle (in order of most to least dysregulated)

## Version 1 09/26/2023

In-Press Preview

## Version 2 11/08/2023

Electronic publication
