# Supplementary material for: Dimethyl fumarate modulates the dystrophic disease program following short-term treatment
Source: JCI Insight. 2023 Nov 8;8(21):e165974. doi: 10.1172/jci.insight.165974 (PMC10721277; doi:10.1172/jci.insight.165974)
Supplement: Supplemental data [file jciinsight-8-165974-s233.pdf]

SUPPLEMENTAL MATERIAL

Dimethyl fumarate modulates the dystrophic disease program following short-term treatment

Cara A. Timpani*[1,2,3], Stephanie Kourakis*[1,2], Danielle A. Debruin[1,2], Dean G. Campelj[1], Nancy Pompeani[1,4], Narges Dargahi[1], Angelo P. Bautista[5], Ryan M. Bagaric[1,2], Elya J. Ritenis[6], Lauren Sahakian[1,3], Didier Debrincat[6], Nicole Stupka[3,2,1], Patricia Hafner[7], Peter G. Arthur[5], Jessica R. Terrill[5], Vasso Apostolopoulos[1,2], Judy B. de Haan[8,9,10,11,12], Nuri Guven[13], Dirk Fischer[7], Emma Rybalka*[1,2,3,7#]

[1]Victoria University, Institute for Health and Sport (IHeS), Melbourne, Victoria, Australia

[2]Australian Institute for Musculoskeletal Science (AIMSS), St Albans, Victoria, Australia

[3]The University of Melbourne, Department of Medicine – Western Health, Melbourne Medical School, St Albans, Victoria, Australia

[4]Florey Institute of Neuroscience and Mental Health, Heidelberg, Victoria, Australia

[5]The University of Western Australia, School of Molecular Sciences, Perth, Western Australia, Australia

[6]Victoria University, College of Health and Biomedicine, Melbourne, Victoria, Australia

[7]University Children's Hospital of Basel (UKBB), Division of Neuropaediatrics and Developmental Medicine, Basel, Switzerland

[8]Baker Heart and Diabetes Institute, Oxidative Stress Laboratory, Basic Science Domain, Melbourne, Victoria, Australia

[9]Department of Immunology and Pathology, Central Clinical School, Monash University, Melbourne, Victoria, Australia

[10]Department of Physiology, Anatomy and Microbiology, La Trobe University, Melbourne, Australia

[11]Faculty of Science, Engineering and Technology, Swinburne University, Melbourne Australia

[12]Baker Department of Cardiometabolic Health, University of Melbourne, Parkville, Australia

[13]University of Tasmania, School of Pharmacy and Pharmacology, Hobart, Tasmania, Australia

D.A.D. is now affiliated with Department of Biochemical and Physiological Sciences, Faculty of Health and Medical Sciences, University of Surrey, Guildford, Surrey, England

D.G.C. is now affiliated with: Centenary Institute, Biology of Ageing Laboratory, Sydney, New South Wales, Australia

E.J.R. is now affiliated with: Swinburne University of Technology, Department of Health Sciences and Biostatistics, Melbourne, Australia

*Authors contributed equally, order of co-first authors are assigned alphabetically (first name)

[#]Correspondence: emma.rybalka@vu.edu.au; Tel.: +61-3-8395-8226; Institute for Health and Sport, Victoria University, PO Box 14428, Melbourne City MC, Victoria, Australia, 8001.

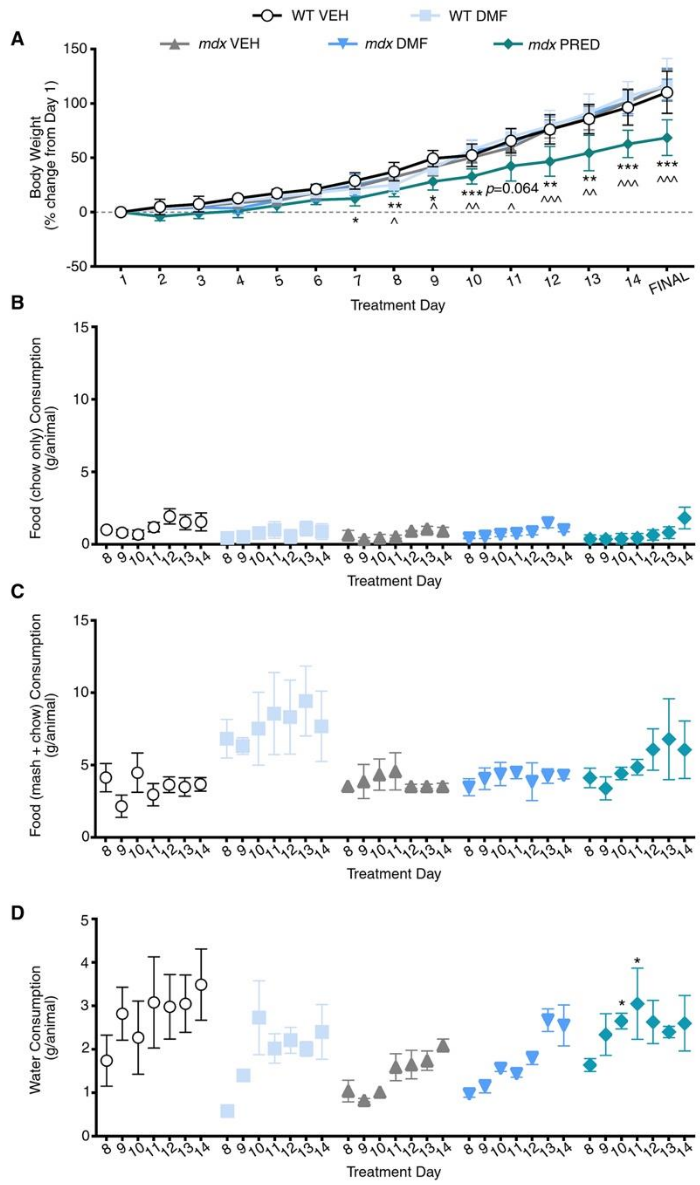

Supplementary Figure 1. Daily dimethyl fumarate (DMF) treatment is safe in juvenile mice. (A) Body weight was monitored daily over the treatment period with **(B-C)** food and **(D)** water consumption monitored from day 8 of treatment. Panel A *n* mice: WT VEH=11, WT DMF=9, *mdx* VEH=10, *mdx* DMF=9, *mdx* PRED=9. Panel B-D *n* boxes: WT VEH=4, WT DMF=3, *mdx* VEH=2, *mdx* DMF=4, *mdx* DMF=3. Treatment effect: **p*<0.05, ***p*<0.01, ****p*<0.001; PRED versus DMF effect: ^*p*<0.05, ^^*p*<0.01, ^^^*p*<0.001.

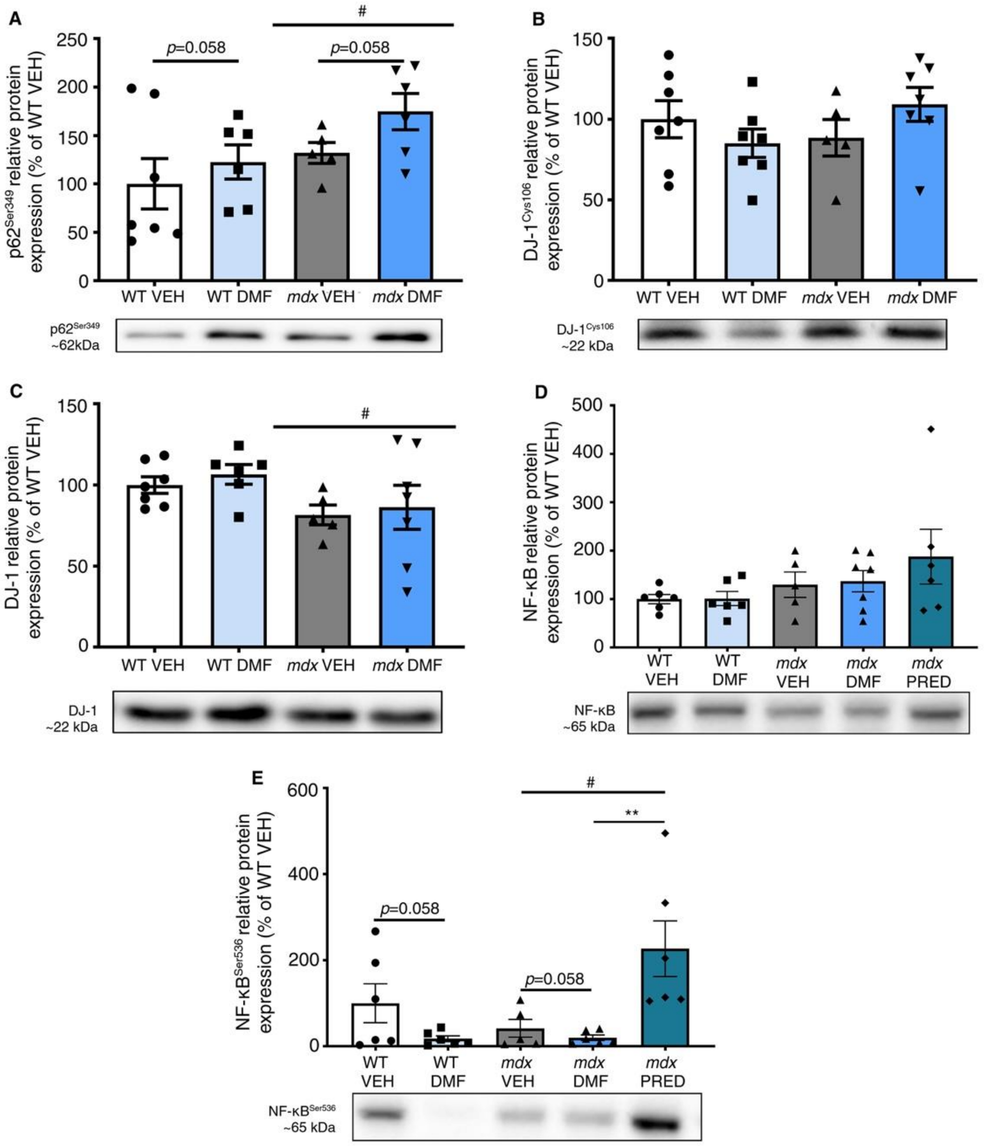

Supplementary Figure 2: Protein expression of other proteins involved in nuclear erythroid 2-related factor 2 (Nrf2) activation and inflammation. (A) Phosphorylated (Serine 329) sequestosome 1 (p62) , **(B)** phosphorylated and **(C)** total protein deglycase/Parkinson's protein 7 (DJ-1), **(D)** total and **(E)** phosphorylated (Serine 536) nuclear factor kappa B (NF-κB) protein was quantitated via western blot. Data are mean ± SEM and *n* are indicated by individual data points. Statistical significance was tested by two-factor (genotype and DMF treatment) and one-factor (*mdx* treatment) ANOVA. Treatment effect: $^{**}p<0.01$; genotype effect: $^{\#}p<0.05$.

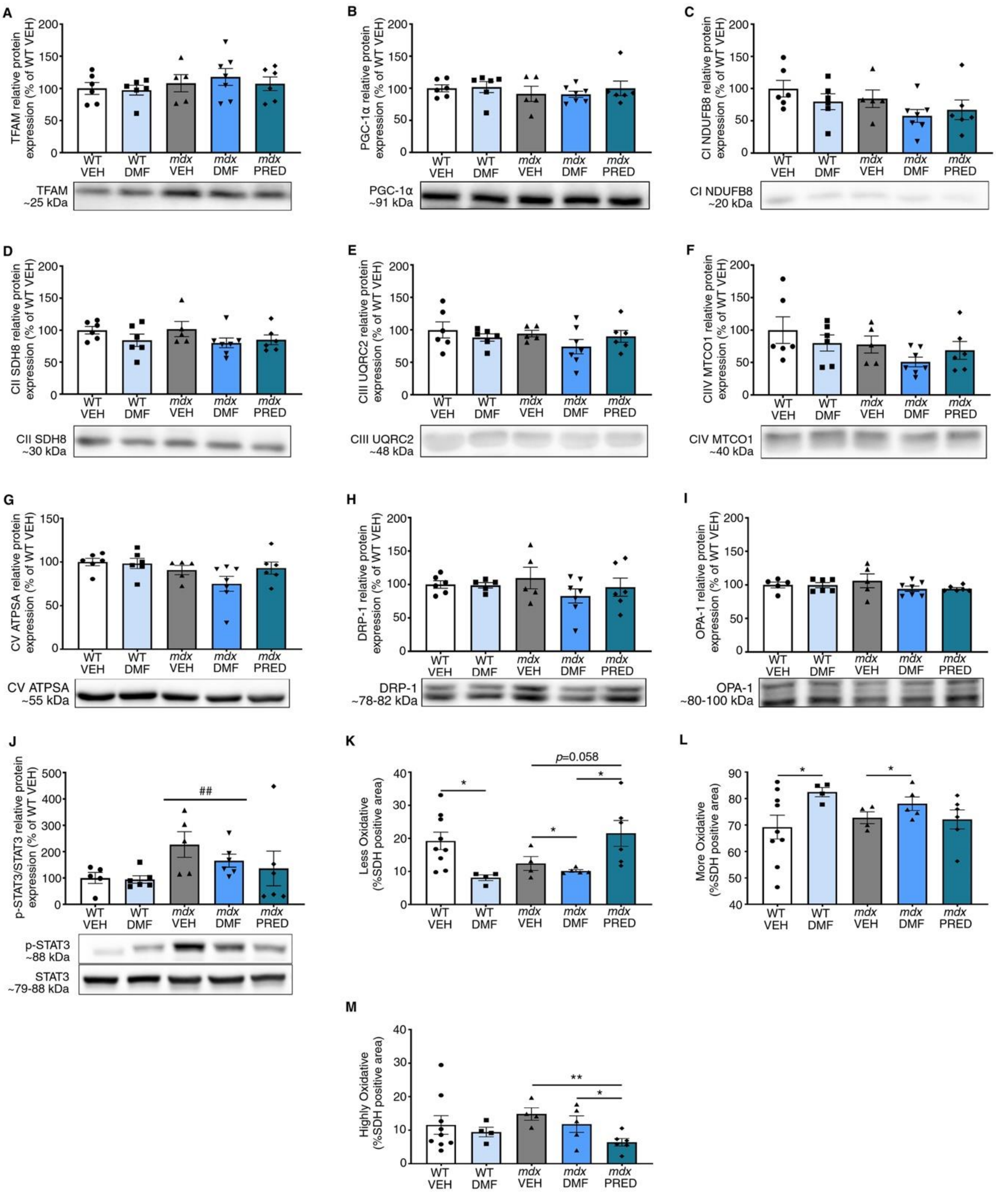

Supplementary Figure 3. Dimethyl fumarate (DMF) does not alter mitochondrial protein expression but does augment the oxidative capacity of *mdx* muscles. (A) Mitochondrial transcription factor A (TFAM), **(B)** peroxisome proliferator-activated receptor-gamma coactivator-1α (PGC-1α), **(C-G)** mitochondrial complex subunits I-V, **(H)** dynamin-related protein 1 (DRP-1) (I) OPA-1 and **(J)** the ratio of phosphorylated to total signal transducers and activators of transcription 3 (STAT3) protein expression was quantitated via western blot. The oxidative fibre type composition of tibialis anterior (TA) was assessed via succinate dehydrogenase (SDH) activity staining **(K-M)**. Data are mean $\pm$ SEM and *n* are indicated by individual data points. Statistical significance was tested by two-factor (genotype and DMF treatment) and one-factor (*mdx*

treatment) ANOVA. Treatment effect: **p*<0.05, ***p*<0.01; genotype effect: $^{\#\#}$*p*<0.01.

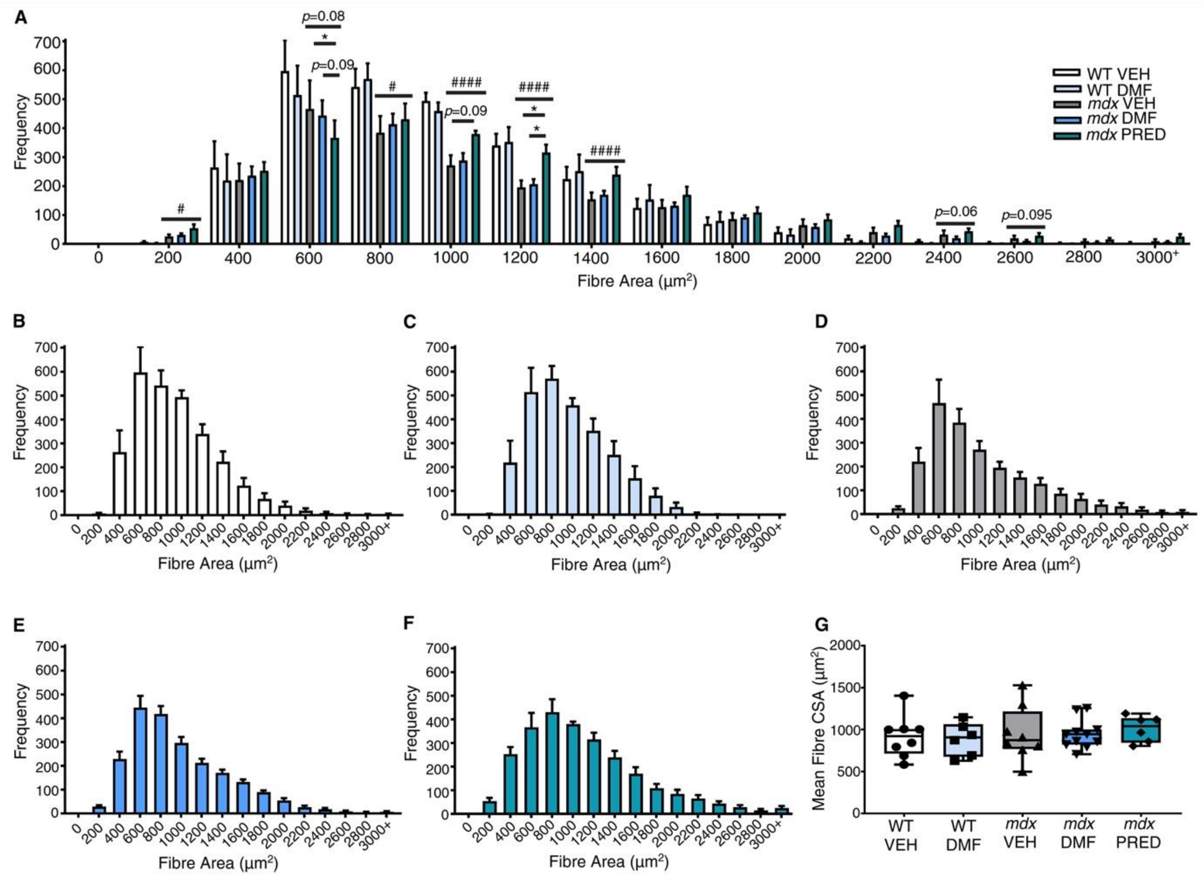

Supplementary Figure 4. Dimethyl fumarate (DMF) does not impact the cross-sectional area (CSA) of tibialis anterior muscle fibres. (A-F) Fibre size distributions and **(G)** summary fibre CSA data of tibialis anterior (TA). Data are mean $\pm$ SEM and *n* are Panel A-F *n*: WT VEH=8, WT DMF=6, *mdx* VEH=8, *mdx* DMF=11, *mdx* PRED=6; Panel H indicated by individual data points. Statistical significance was tested by two-factor (genotype and DMF treatment) and one-factor (*mdx* treatment) ANOVA. Treatment effect: **p*<0.05; genotype effect: $^{\#}p<0.05$, $^{\#\#\#}p<0.001$, $^{\#\#\#\#}p<0.0001$.

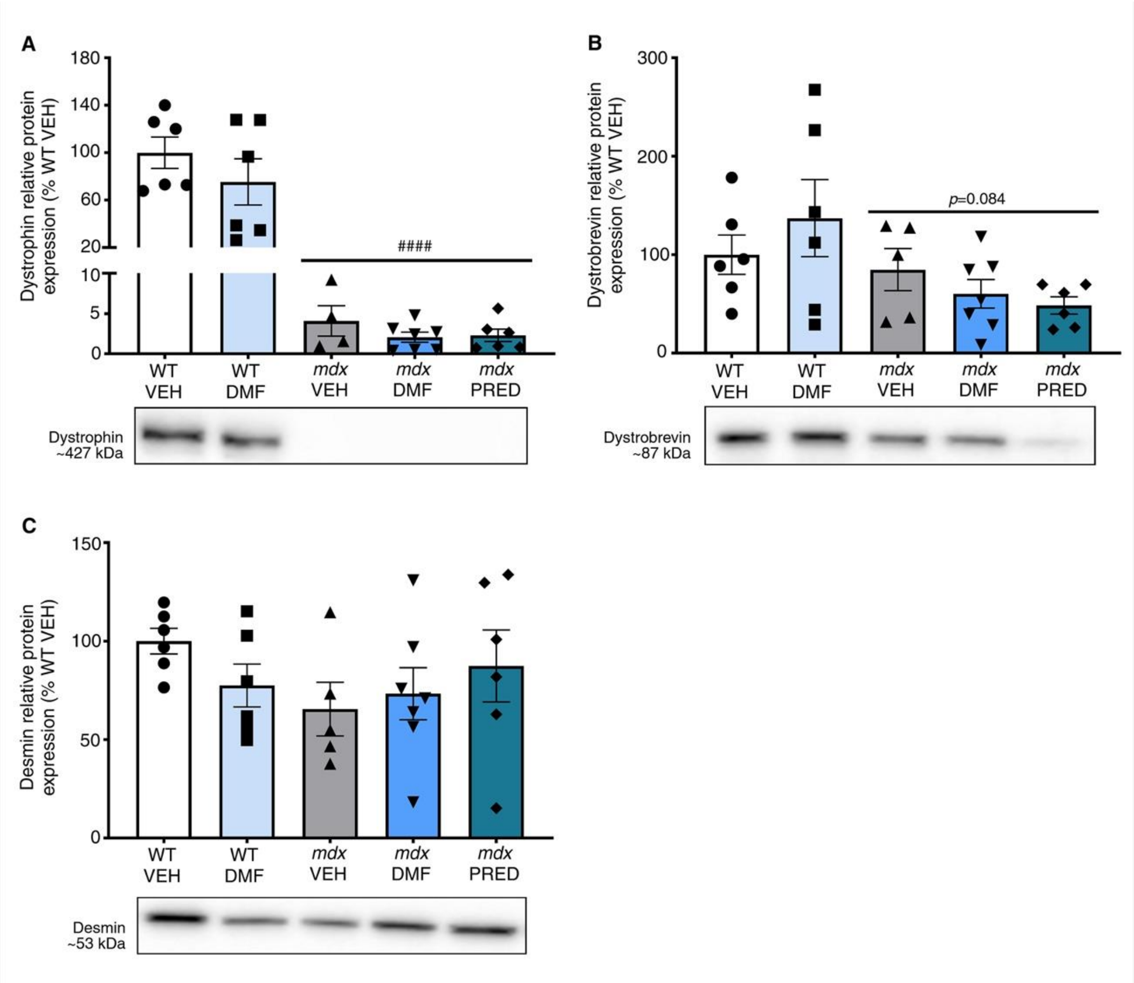

Supplementary Figure 5. Dimethyl fumarate (DMF) does not alter the expression of the cytoskeletal proteins dystrophin, dystrobrevin or desmin. The protein expression of **(A)** dystrophin, **(B)** dystrobrevin and **(C)** desmin was quantitated via western blot. Data are mean ± SEM and *n* are indicated by individual data points. Statistical significance was tested by two-factor (genotype and DMF treatment) and one-factor (*mdx* treatment) ANOVA. Genotype effect: $^{\#\#\#\#}p<0.0001$.

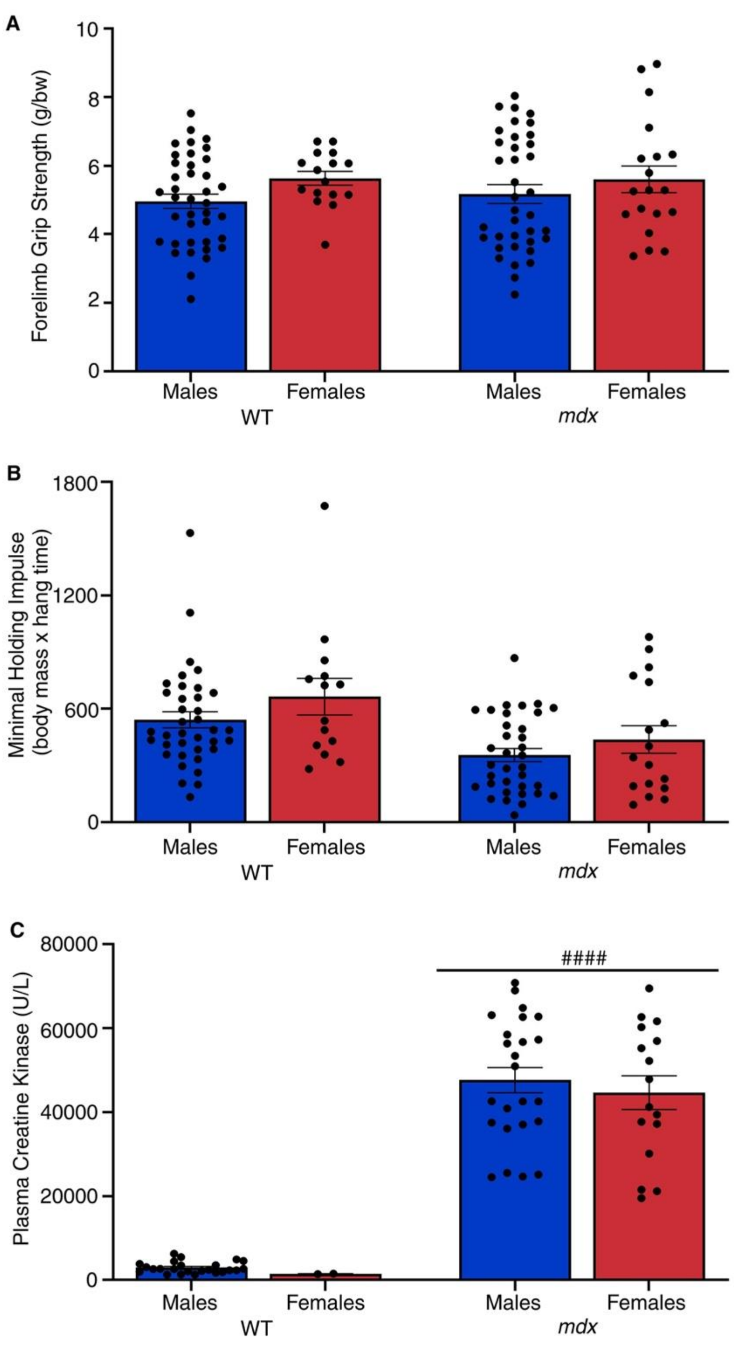

Supplementary Figure 6. Comparable phenotype between male and female juvenile *mdx* mice. There were no significant differences in **(A)** forelimb grip strength, **(B)** minimal holding impulse or **(C)** plasma creatine kinase (CK) levels between juvenile male and female *mdx* mice. Data are mean ± SEM and *n* are indicated by individual data points. Statistical significance was tested by two-factor (genotype and sex). Genotype effect: $^{####}p$<0.0001.

Supplementary Table 1: Effect of genotype and treatments on organ mass of wild-type (WT) and dystrophic *mdx* mice. Data are mean ± SEM. Statistical significance was tested by two-factor (genotype and DMF treatment) and one-factor (*mdx* treatment) ANOVA: $^{\#}p<0.05$ and $^{\#\#\#}p<0.005$ different from WT VEH; $^{***}p<0.005$ treatment effect, $^{\wedge\wedge\wedge}p<0.005$ PRED different from DMF.

	WT		*mdx*		
	VEH ($mg^{-1}.g\ bw^{-1}$)	DMF ($mg^{-1}.g\ bw^{-1}$)	VEH ($mg^{-1}.g\ bw^{-1}$)	DMF ($mg^{-1}.g\ bw^{-1}$)	PRED ($mg^{-1}.g\ bw^{-1}$)
Heart	4.71 ± 0.311 (*n*=10)	5.19 ± 0.099 (*n*=8)	5.29 ± 0.103 (*n*=9)	5.41 ± 0.062 (*n*=6)	5.34 ± 0.089 (*n*=6)
Lungs	8.66 ± 0.361 (*n*=9)	8.18 ± 0.228 (*n*=7)	$9.95 \pm 0.300^{\#}$ (*n*=9)	9.427± 0.346 (*n*=6)	9.99 ± 0.442 (*n*=7)
Liver	55.26 ± 1.16 (*n*=9)	51.37 ± 1.15 (*n*=7)	$46.05 \pm 1.59^{\#\#\#}$ (*n*=9)	$55.32 \pm 1.20^{***}$ (*n*=7)	52.70 ± 3.63 (*n*=7)
Spleen	5.33 ± 0.203 (*n*=9)	4.86 ± 0.248 (*n*=8)	4.86 ± 0.031 (*n*=9)	4.94 ± 0.190 (*n*=7)	$2.98 \pm 0.227^{***\wedge\wedge\wedge}$ (*n*=7)
Kidneys	6.67 ± 0.176 (*n*=10)	6.99 ± 0.158 (*n*=8)	7.02 ± 0.194 (*n*=9)	7.27 ± 0.108 (*n*=7)	7.13 ± 0.133 (*n*=7)

Supplementary Table 2: Contractile function characteristics of extensor digitorum longus (EDL) and soleus muscle from wild-type (WT) and *mdx* mice treated with vehicle (VEH), dimethyl fumarate (DMF) or prednisone (PRED). Data are mean ± SEM. Statistical significance was tested by two-factor (genotype and DMF treatment) and one-factor (*mdx* treatment) ANOVA: $^{\#}p<0.05$, $^{\#\#}p<0.01$ (*mdx* VEH v WT VEH); $^{*}p<0.05$, $^{**}p<0.01$, $^{****}p<0.0001$ (VEH v DMF); $^{\wedge}p<0.05$, $^{\wedge\wedge\wedge}p<0.001$, $^{\wedge\wedge\wedge\wedge}p<0.0001$ (DMF v PRED).

	**EDL**					**Soleus**				
	**WT**		***mdx***			**WT**		***mdx***		
	**VEH**	**DMF**	**VEH**	**DMF**	**PRED**	**VEH**	**DMF**	**VEH**	**DMF**	**PRED**
Muscle mass (mg)	4.98 ± 0.51 (*n*=10)	4.32 ± 0.37 (*n*=8)	4.39 ± 0.14 (*n*=10)	4.45 ± 0.28 (*n*=8)	5.78 ± 0.47 (*n*=6)	5.95 ± 0.56 (*n*=10)	4.78 ± 0.36** (*n*=8)	6.46 ± 0.41 (*n*=10)	3.59 ± 0.55** (*n*=8)	6.34 ± 0.15^ (*n*=5)
Optimum length (L_o; mm)	7.62 ± 0.66 (*n*=8)	5.57 ± 0.19**** (*n*=7)	9.67 ± 0.23$^{\#}$ (*n*=8)	5.85 ± 0.37**** (*n*=7)	10.20 ± 0.87^^^^ (*n*=5)	7.50 ± 0.69 (*n*=8)	5.17± 0.15* (*n*=6)	10.67 ± 0.14$^{\#}$ (*n*=8)	4.60 ± 0.22**** (*n*=5)	8.70 ± 0.91^^^ (*n*=5)
Absolute (maximal isometric) force (mN)	174.75 ± 26.01 (*n*=8)	81.02 ± 14.11** (*n*=7)	39.32 ± 5.84$^{\#\#}$ (*n*=8)	92.22 ± 11.67$^{\#\#}$ (*n*=7)	98.64 ± 21.76 (*n*=5)	118.05 ± 23.36 (*n*=8)	94.22 ± 12.52 (*n*=6)	38.57 ± 5.63$^{\#\#}$ (*n*=7)	38.87 ± 8.42$^{\#\#}$ (*n*=5)	52.25 ± 7.38 (*n*=6)

Supplementary Table 3: Effect of dimethyl fumarate (DMF) versus prednisone (PRED) treatment on normally expressed genes (NEGs) involved in extracellular matrix composition and cell adhesion in *mdx* compared to wild-type (WT) muscle (in order of most to least effected by PRED). Drug effect on NEG criteria is >1.5-fold regulation, $p<0.05$ from reference groups (*mdx* DMF and PRED v *mdx* VEH). Key: BMC: basement membrane constituents; CAM: cell adhesion molecule; ECMM: extracellular matrix molecules. Bolded data represent significant treatment effects: Blue shading indicates DMD disease seed genes. *n*=4/group where each *n* is equivalent to pooled mRNA for *n*=2 mice.

NEG (Mdx v WT)		***DMF Effect***		***PRED Effect***		***Class***
Gene	**Fold Reg.**	***Fold Reg.***	***p***	***Fold Reg.***	***p***	
Mmp13	1.82	-1.90	0.176	↓ **3.36**	**	*ECMM*
Tnc	3.23	↓ **2.21**	*	↓ **4.77**	****	*Other*
Mmp3	1.94	↓ **2.23**	***	↓ 2.54	0.218	*ECMM*
Selp	1.39	**-1.78**	*	↓ **2.47**	*	*CAM*
Itgb2	3.81	-1.82	0.21	↓ 2.21	0.071	
Itgal	1.82	-1.12	0.438	↓ **2.19**	****	
Sele	-1.43	-1.43	0.132	↓ **2.18**	**	
Adamts8	-1.76	1.39	0.21	**2.10**	*	*ECMM*
Icam1	2.03	-1.33	0.061	↓ **2.08**	****	*CAM*
Sell	-1.24	-1.22	0.295	↓ 2.05	0.064	
Postn	2.82	1.17	0.46	↓ **2.01**	*	*Other*

Cd44	2.50	-1.60	0.883	**-1.85**	**	
Thbs1	1.74	-1.25	0.407	**-1.75**	**	
Itga3	-1.50	-1.29	0.103	**-1.73**	*	
Entpd1	-1.01	-1.11	0.449	**-1.60**	**	
Col6a1	-1.14	1.16	0.229	**-1.41**	*	
Sgce	-1.55	**1.27**	*	**-1.41**	*	
Itga5	1.26	**-1.35**	*	**-1.37**	*	
Fn1	1.27	1.05	0.631	**-1.32**	*	
Ctgf	1.41	**-1.80**	*	-1.05	0.685	
Col5a1	-1.17	**1.20**	*	1.11	0.533	
